# Phylogeography and Population Genetics of *Rosa chinensis* var. s*pontanea* and *R. lucidissima* Complex, the Important Ancestor of Modern Roses

**DOI:** 10.3389/fpls.2022.851396

**Published:** 2022-05-20

**Authors:** Hongying Jian, Ling Zhao, Hao Zhang, Changle Ma, Qigang Wang, Huijun Yan, Xianqin Qiu, Ningning Zhou, Ting Zhang

**Affiliations:** ^1^National Engineering Research Center for Ornamental Horticulture/Flower Research Institute, Yunnan Academy of Agricultural Sciences, Kunming, China; ^2^School of Landscape Architecture and Horticulture Science, Southwest Forestry University, Kunming, China

**Keywords:** conservation, genetic variation, population history, refugia, evolutionary mechanism, species boundary

## Abstract

*Rosa chinensis* var. *spontanea* and *R. lucidissima* complex are the morphologically very similar key ancestors of modern roses with high importance in rose research and breeding. Although widely distributed in subtropical central and southwestern China, these two taxa are highly endangered. We sampled a total of 221 specimens and 330 DNA samples from 25 populations across the two taxa's whole range. Leaf morphological traits were compared. Two chloroplast DNA intergenic spacers (*trn*G*-trn*S, *pet*L*-psb*E) and ITS were used for population genetics and phylogenetic study to delimit the boundary between the two taxa, assess the genetic variation, uncover the possible evolutionary mechanism responsible for the differentiation within the complex, and make the conservation recommendations. The complex exhibited high levels of genetic variation (*h*_TcpDNA_ = 0.768, *h*_TITS_ = 0.726) and high population differentiation even over small geographic distance. We suggest *R. chinensis* var. *spontanea* and *R. lucidissma* be treated as independent taxa, and the northern populations around and within the Sichuan Basin being *R. chinensis* var. *spontanea*, having broader leaflets and paler full-blooming flowers, while those in the middle and southern Yunnan-Guizhou Plateau and the adjacent regions being *R. lucidissma*, having narrower leaflets and darker full-blooming flowers. Transitional areas between the southeastern Sichuan Basin and northeastern Guizhou are the contact or the hybridization zone of the two taxa. Ancestral haplotypes of the complex (*R. lucidissma*) evolved at about 1.21–0.86 Mya in southeastern Yunnan-Guizhou Plateau and its adjacent regions and survived there during the Quaternary Oscillation. Ancestral haplotypes of *R. chinensis* var. *spontanea* deviated from *R. lucidissma* at about 0.022–0.031 Mya at the transitional areas (Daloushan and Wulingshan Mountains) between the northeastern edge of Yunnan-Guizhou Plaeteau and the southeastern border of Sichuan Basin, where they survived the LGM. The evolution of the complex included spatial isolation and inter-species hybridization. The complex's endangered status might be the result of over-exploitation for its ornamental and medical value, or due to reforestation of some originally open habitats. We provide specific recommendations for the two taxa's *in situ* and *ex situ* conservation.

## Introduction

Phylogeographical studies utilizing genetic markers having phylogenetic signals such as cpDNAs and nrITS and analyzing the spatial distribution of haplotypes across species or species complex ranges, allow for verifying taxonomy at various taxonomic levels. Besides, such studies can unravel historical processes that led to the formation of species or affected their distribution (e.g., post-glacial migrations) and identify potential glacial refugia (Avise, 2000, 2004; Thaw et al., 2019). Refugia, i.e., locations where species have persisted during long-term climatic changes due to their eco-climatic stability, have high conservation value, so it is very important to identify precise refugial locations (Médail and Diadema, 2009; Hampe et al., 2013; Volis, 2019). In China, the area within ~34°N to 22°N (Wu, 1980; Qiu et al., 2011) is considered a Pleistocene refugium for many ancient and relict species. However, within this area, multiple isolated refugia have been revealed, mostly in mountain regions (see Zhang et al., 2016; Deng et al., 2019 and references therein). Among them, the Sichuan Basin, which is part of the boundary between the Sino-Japanese Forest subkingdom and the Sino-Himalayan Forest subkingdom (Wu and Wu, 1996), played an important role in the formation and evolution of many plant lineages (Gao et al., 2007; Mitsui et al., 2008; Qiu et al., 2009; Guan et al., 2010; Ma et al., 2015).

**Figure 1:**
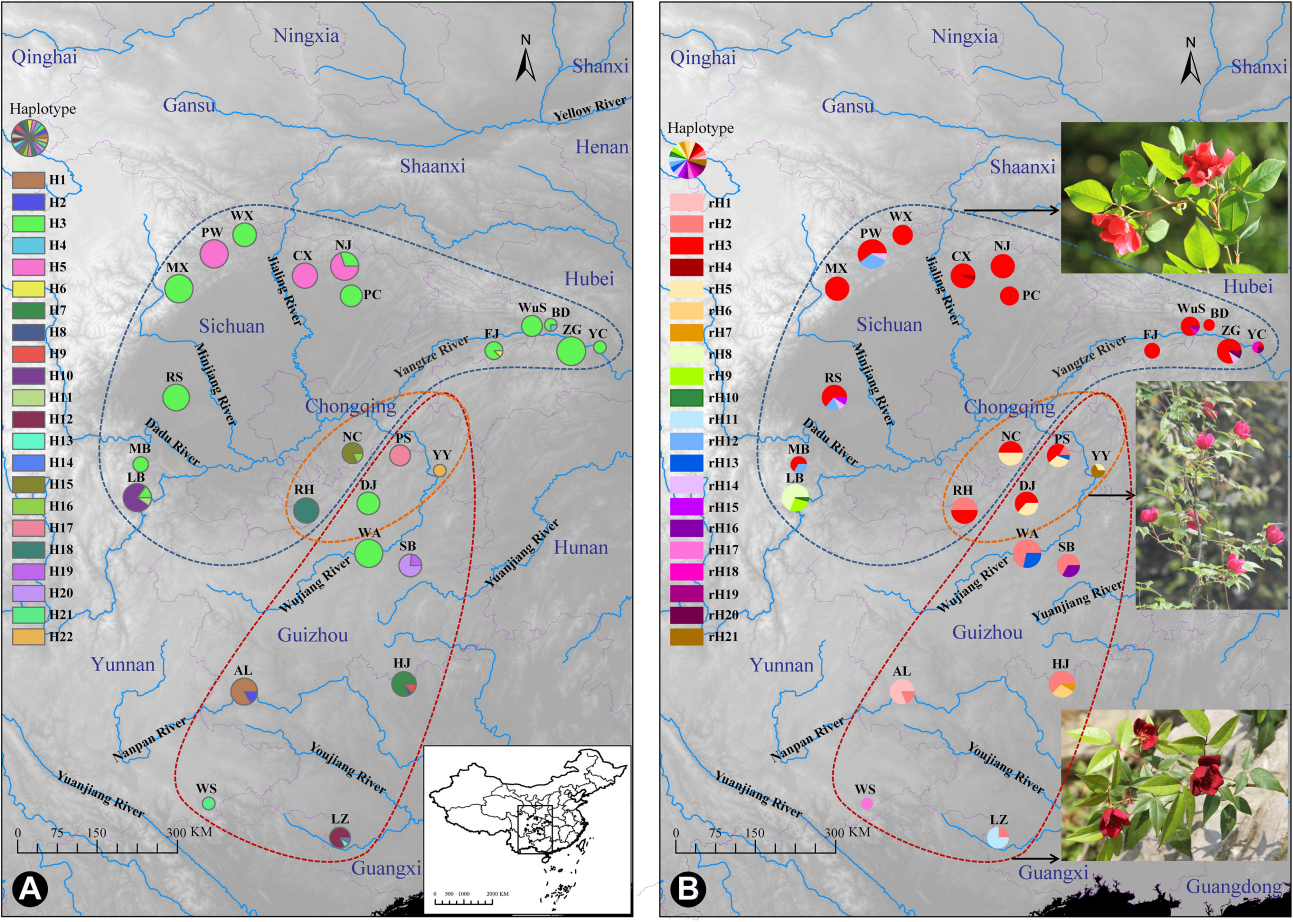
Distribution of cpDNA haplotypes **(A)** and ITS ribotypes **(B)** of *Rosa chinensis* var. *spontanea* (area encircled by the blue curve) and *R. lucidissima* (area encircled by the red curve). Pie charts show the frequency of haplotypes/ribotypes in each population. The approximate location of the complex's distribution was shown in **(A)**. The orange curve shows the transitional zones of the two taxa. The photos of each taxa and the plant in the transitional zones were shown close to their geographical distribution, respectively, in **(B)**.

**Figure 2:**
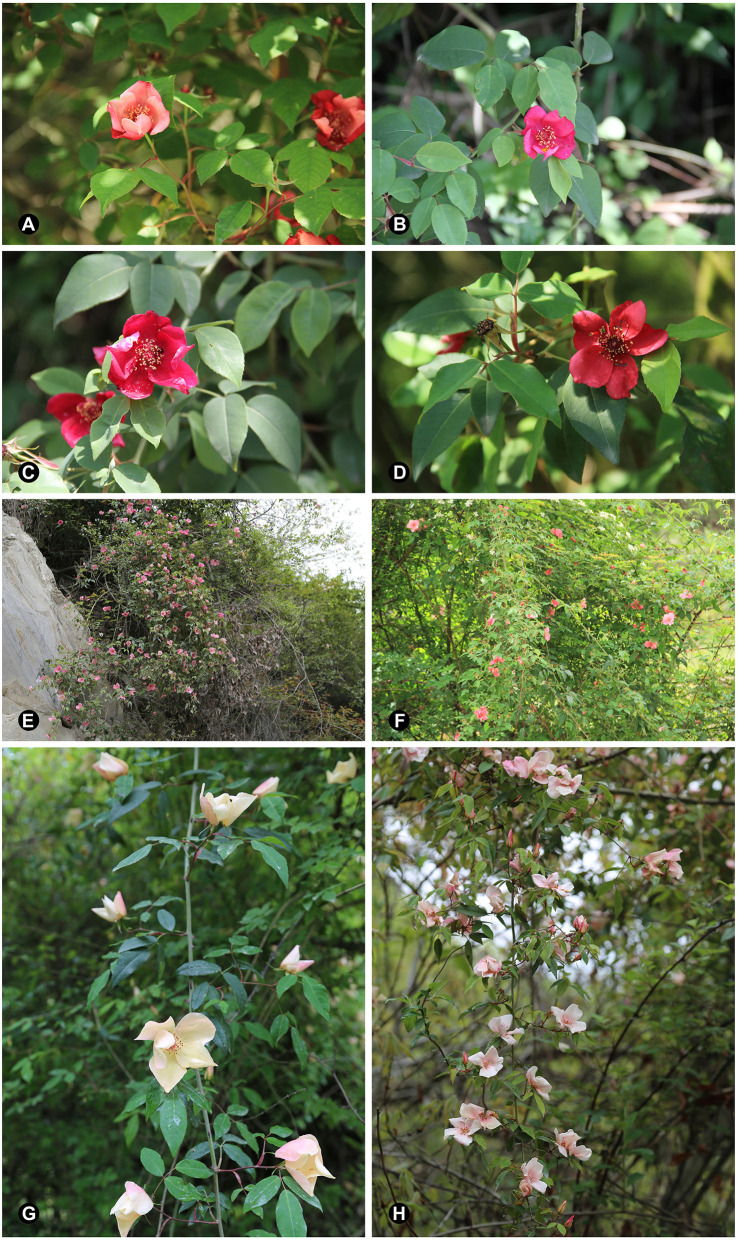
Change of petal color different opening stage and morphological variation within population MX. **(A)** Initial blooming stage. **(B)** Full-blooming stage. **(C)** Post-blooming stage. **(D)** Wither stage. **(E)** Individuals with pink flowers. **(F)** Individuals with crimson flowers. **(G)** Individuals with creamy white flowers. **(H)** Individuals with pinkish-white flowers.

Species are a fundamental unit of biology, but discerning species boundaries among closely related taxa is a difficult task. The genus *Rosa* L. (Rosaceae) comprises about 150–200 species widely distributed in the temperate and subtropical northern hemisphere, and about half of the rose species occur in Asia, especially in China (Ku, 1985; Ku and Robertson, 2003). Species of this genus are difficult to identify due to similar morphology because of naturally occurring hybridization (Fougère-Danezan et al., 2014) and polyploidization (De Riek et al., 2013). Chinese roses, especially natural or cultivated varieties of both *R. chinensis* Jacq. and *R. odorata* (Andr.) Sweet have been contributing characteristic of recurrent flowering, tea scent, and multiple floral colors to modern roses (Hurst, 1941; Wylie, 1954; Scalliet et al., 2008; Raymond et al., 2018). The wild type of *R. chinensis, R. chinensis* var. *spontanea* (Rehder et. Wilson) Yü et Ku (Ku, 1985; Ku and Robertson, 2003) from sect. *Chinenses*, is the maternal parent of *R. chinensis* and the possible paternal parent of *R. odorata* (Meng et al., 2011). Thus, *R. chinensis* var. *spontanea* is one of the key ancestors of modern roses. *Rosa lucidissima* Lévl., also from sect. *Chinenses*, are so similar in morphology to *R. chinensis* var. *spontanea* that it was thought to be the state of *R. chinensis* var. *spontanea* by both Henry and Wilson (Rix, 2005). Both taxa are diploid (Akasaka et al., 2002; Cao et al., 2021), have shiny deep green leaves of 5–11 cm with 3–5 leaflets, solitary single flowers, and ovoid, obovoid, or pyriform glabrous hips fruiting from May to September. *Rosa chinensis* var. *spontanea* is an “erect shrub”. Its rachis and petiole are sparsely prickly and glandular-pubescent, leaflets are “broadly ovate or ovate-oblong” and flowers are red. By contrast, *R. lucidissima* is a “climbing shrub” having rachis and petiole that are shortly prickly and sparsely glandular-pubescent, leaflets are “oblong-ovate or long elliptic” and pale green abaxially, and flowers are purple-red (Ku, 1985; Ku and Robertson, 2003; Figure 1). However, it is difficult to tell these two taxa apart in the wild, and it is common when specimens with the same collection ID are identified in one herbarium as *R. chinensis* var. *spontanea* and in another as *R. lucidissma*. In the field investigations, all the plants (and even in the place where the type specimen of *R. chinensis* var. *spontanea* was collected) are climbing shrubs. Plants flower from early March to early May with extraordinary petal color variation (Rix, 2005 and personal observations in the field). During flower opening, the petal color is changing from pink to purple (Figures 2A–D), and in populations in northern Sichuan, e.g., Pingwu county and Maoxian county, the flower colors of different plants varies from creamy white to dark crimson (Figures 2E–H). It is still not clear how much of this variation is due to phenotypic plasticity and how much is the result of genetic differentiation. Population level studies seem more effective for tracking evolutionary history and circumscribing close species (Joly et al., 2006; Liu et al., 2012; Sun et al., 2014). We treat *R. chinensis* var. *spontanea* and *R. lucidissima* as a complex and sampled according to populations for further study.

According to the specimen records, *R. chinensis* var. *spontanea* and *R. lucidissima* occur in subtropical central and Southwestern China, mainly on the borders of the Sichuan Basin, further north to the south of Gansu, further south to the east of Yunnan-Guizhou Plateau, Jiuwan Mountains and Shiwan Mountains in Guanxi Zhuang Autonomous Region and south of Nanling Mountains in Guangdong provinces (Figure 1). Plants have not only decorative but also medicinal value (Ye, 2015; Zhao et al., 2019) and demands for their roots are increasing with the development of public transportation and information allowing horticultural and pharmaceutical companies to reach the remote rural areas to buy the plants either for gardening or for medicine. Not surprisingly, the populations of these taxa are rapidly decreasing in size or completely disappearing. Meng et al. (2011) failed to collect any plants during their field expedition to the specimen-recorded locations. Only 14 individuals were found in Beichuan and Pingwu counties in NW Sichuan by Cui et al. (2016). *Rosa chinensis* var. *spontanea* and *R. lucidissima* have been listed as “endangered” and “critically endangered”, respectively, in a recent report on biodiversity (Qin et al., 2017). In the most recently published List of National Key Protected Wild Plants (http://www.forestry.gov.cn/main/3954/20210908/163949170374051.html), both taxa are evaluated as Rank II. The high importance of the above two taxa in rose research and breeding, and conservation concerns necessitate understanding the extent and structure of their genetic diversity. In our study, we sampled *R. chinensis* var. *spontanea* and *R. lucidissima* across their whole range, and used two chloroplast DNA regions and ITS to (1) delimit the boundary between the two taxa and uncover the possible evolutionary mechanism responsible for the differentiation within the complex; (2) analyze the extent and structure of the genetic variation of the complex; (3) identify the possible refugia during the glaciation period and the population history of the complex; and (4) make the conservation recommendations.

## Materials and Methods

### Plant Material Sampling

A total of 330 individuals (4 to 21 per population depending on the population size) were sampled from 25 populations, covering the whole geographical distribution of the complex (Table 1). Fresh young leaves were collected and dried in silica gel during field expeditions. A total of 221 specimen (1–20 per population) were collected for morphological trait study. The latitude, longitude, and altitude of each population were recorded. *Rosa rugosa* (sect. *Cinnamomeae*), *R. woodsii* (sect. *Carolinae*), *R. mairei* (sect. *Pimpinellifoliae*), *R. banksiae* (sect. *Banksianae*), and *R. bracteata* (sect. *Bracteatae*) were used as outgroups in the phylogenetic analysis. Fresh leaves of these species were collected from the Yunnan Provincial Flower Gemplasm Resources Nursery in the Flower Research Institute, Yunnan Academy of Agricultural Sciences in Kunming, China.

**Table 1:** Details of the sample locations, sample sizes, haplotype frequencies, haplotype and nucleotide diversity based on cpDNA fragments and ITS.

**Pop. ID**	**Province**	LON^*^	LAT^*^	**ALT (m)**	**Taxa^#^**	**Individuals**	**cpDNA (*****trnS-trn*****G**, ***pet*****L*****-psb*****E)**			**ITS**			
							**Haplotype frequencies**	**Haplotype diversity, HD (SD)**	**Nucleotide diversity Pi (SD) × 10^**−3**^**	**Number of sequence**	**Haplotype frequencies**	**Haplotype diversity, Hd (SD)**	**Nucleotide diversity, Pi (SD) × 10^**−3**^**
BD	Hubei	110.4	31.2	608	*R.C.S*	4	H3 (3), H4 (1)	0.50 (0.27)	0.20 (0.11)	4	rH3 (4)	0 (0)	0(0)
CX	Sichuan	106.3	32.0	494	*R.C.S*	16	H5(16)	0 (0)	0	21	rH3 (20), rH4 (1)	0.10 (0.08)	0.89 (0.79)
FJ	Chongqing	109.5	30.8	370	*R.C.S*	8	H3 (7), H6 (1)	0.25(0.18)	0.10 (0.07)	8	rH3 (8)	0 (0)	0 (0)
LB	Sichuan	103.4	28.3	1163	*R.C.S*	20	H3 (3), H10 (15), H11 (2)	0.43 (0.12)	0.19 (0.06)	26	rH8 (18), rH9 (7), rH10 (1)	0.47 (0.09)	1.05 (0.38)
MB	Sichuan	103.5	28.8	1046	*R.C.S*	6	H3 (6)	0 (0)	0	9	rH3 (6), rH12 (3)	0.50 (0.13)	5.46 (1.40)
MX	Sichuan	104.1	31.8	1134	*R.C.S*	20	H3 (20)	0 (0)	0	21	rH3 (20)	0	0
NC	Chongqing	107.1	29.0	300	*R.C.S*	11	H15 (9), H16 (2)	0.33 (0.15)	0.26 (0.12)	20	rH3 (10), rH5 (10)	0.53 (0.04)	5.75 (0.4)
NJ	Sichuan	106.9	22.5	585	*R.C.S*	20	H3 (6), H5 (14)	0.44 (0.09)	0.72 (0.14)	20	rH3 (20)	0	0
PC	Sichuan	107.0	31.7	390	*R.C.S*	12	H3 (12)	0 (0)	0	12	rH3 (12)	0	0
PW	Sichuan	104.7	32.4	850	*R.C.S*	20	H5 (20)	0 (0)	0	30	rH3 (18), rH12 (10), rH14 (2)	0.54 (0.06)	5.62 (0.51)
RH	Guizhou	106.3	28.1	591	*R.C.S*	16	H18 (16)	0 (0)	0	24	rH2 (12), rH3 (12)	0.52 (0.03)	6.51 (0.38)
RS	Chongqing	104.1	30.0	578	*R.C.S*	18	H3 (18)	0 (0)	0	22	rH3 (14), rH12 (4), rH14 (2), rH15 (2)	0.57 (0.11)	5.46 (0.79)
WuS	Chongqing	110.1	31.2	740	*R.C.S*	11	H3 (11)	0 (0)	0	12	rH3 (10), rH18 (1), rH19 (1)	0.32 (0.16)	0.52 (0.28)
WX	Gansu	105.2	32.7	930	*R.C.S*	14	H3 (14)	0 (0)	0	14	rH3 (14)	0	0
YC	Hubei	111.2	30.8	300	*R.C.S*	4	H3 (4)	0 (0)	0	4	rH3 (1), rH18 (2), rH20 (1)	0.83 (0.22)	1.56 (0.53)
ZG	Hubei	110.8	30.8	650	*R.C.S*	21	H3 (21)	0 (0)	0	21	rH3 (17), rH14 (2), rH20 (2)	0.34 (0.12)	2.7 (1.23)
						221		0.62 (0.03)	0.92 (0.06)	267		0.50 (0.04)	5.95 (0.45)
AL	Guizhou	105.2	25.0	1262	*R.L*	18	H1 (15), H2 (3)	0.29 (0.12)	0.12 (0.05)	21	rH1 (17), rH2 (4)	0.32 (0.11)	0.5 (0.17)
DJ	Guizhou	107.3	28.2	740	*R.L*	13	H3 (13)	0 (0)	0	19	rH3 (12), rH5 (7)	0.49 (0.07)	5.36 (0.75)
HJ	Guangxi	107.9	25.1	865	*R.L*	15	H7 (12), H8 (1), H9 (2)	0.36 (0.15)	0.86 (0.36)	24	rH2 (15), rH6(7), rH7(2)	0.54 (0.08)	10.91 (1.3)
LZ	Guangxi	106.9	22.5	423	*R.L*	11	H12 (9), H13 (1), H14 (1)	0.35 (0.17)	1.07 (0.63)	16	rH2 (4), rH11 (12)	0.40 (0.11)	0.62 (0.18)
PS	Chongqing	107.9	29.0	756	*R.L*	11	H17 (11)	0 (0)	0	18	rH2 (3), rH3 (8), rH5 (6), rH13 (1)	0.70 (0.07)	6.35 (0.48)
SB	Guizhou	108.0	27.1	988	*R.L*	13	H19 (3), H20 (10)	0.39 (0.13)	0.16 (0.05)	18	rH2 (12), rH16 (6)	0.47 (0.08)	2.93 (0.51)
WA	Guizhou	107.3	27.3	868	*R.L*	20	H3 (20)	0 (0)	0	28	rH2 (20), rH13 (8)	0.42 (0.08)	4.62 (0.83)
WS	Yunnan	104.6	23.1	892	*R.L*	4	H21 (4)	0 (0)	0	4	rH17 (4)	0	0
YY	Chongqing	108.5	28.7	745	*R.L*	4	H22 (4)	0 (0)	0	6	rH5 (2), rH21 (4)	0.53 (0.17)	4.99 (1.61)
						109		0.89 (0.02)	1.68 (0.1)	154		0.81 (0.02)	7.39 (0.59)
Total						330		0.74 (0.02)	1.95 (0.11)	422		0.72 (0.02)	8.15 (0.25)

# *Nominated according to both the specimen records and morphological characters. R.C.S, R. chinensis var. spontanea; R.L, R. lucidissima*.

* *Only with 1 decimal due to conservation concerns*.

### Molecular Techniques

Total genomic DNA was isolated from the leaves using Plant Axyprep Genomic DNA Kit (Axygen, CA, USA) with some modification to decrease the negative effect of secondary metabolites and epicuticular waxes. From a total of 10 cpDNA regions (*pet*L/*psb*E*, trn*L(UAG*)/rp*l32-F*, trn*H*/psb*A*, trn*DF*/trn*T*, trn*S/*trn*fM*, rp1*20*/rps*12, cpDNA-c*/*cpDNA-f, F71/R1516*, trn*G (UUC)*-trn*S (GCU)*, trn*S*/psb*C), we chose *trn*G (UUC)-*trn*S (GCU) and *pet*L*-psb*E due to their relatively high variation and ease for amplification. The *trn*G (UUC)*-trn*S (GCU) (hereafter referred to as *trn*G*-trn*S) was amplified following the protocol of Shaw et al. (2007). The *pet*L*-psb*E was amplified following that of Shaw et al. (2005). The internal transcribed spacer (ITS) was amplified using the two primers ITS1 and ITS4. The primer sequences of these three fragments were shown in Table 2. Then, the PCR products were purified and sequenced by TsingKe Biological Technology, Beijing, China. For sequence variants appearing in only one or two individuals, amplification and sequencing were repeated to avoid artifacts caused by PCR or sequencing errors. As for those putative hybrids which showed double-peak nucleotide signals in ITS by direct sequencing, the PCR products of ITS were purified with a Gel Extraction Kit (Tsingke, Beijing, China) and then cloned. Cloning of the ITS region was conducted using the pClone 007 versatile simple vector system kit and Trelief™ 5α chemically competent cell (TsingKe, Beijing, China) following the manufacturer's instructions. Plasmids carrying the PCR fragment were then extracted and sequenced. For each PCR product, at least 2 clones were randomly sequenced.

**Table 2:** Primers for the amplification of selected chloroplast intergeneric spacers and ITS.

**Fragment**	**Primer**	**Primer sequence**	**References**
*trn*G*-trn*S	*trn*G	5'-GAATCGAACCCGCATCGTTAG-3'	Wakasugi et al., 1994
	*trn*S	5'-AACTCGTACAACGGATTAGCAATC-3'	
*pet*L*-psb*E	*pet*L	5'-AGTAGAAAACCGAAATAACTAGTTA-3'	Shaw et al., 2005
	*psb*E	5'-TATCGAATACTGGTAATAATATCAGC-3'	
ITS	ITS1	5'-TCCGTAGGTGAACCTGCGG-3'	White et al., 1990
	ITS4	5'-TCCTCCGCTTATTGATATGC-3'	

### Leaf Morphological Traits Measurement

Because some specimens did not have blooming flowers, we focused on the leaflet shapes of both taxa. We randomly selected three mature odd-pinnately compound leaves in each specimen for study. Leaflet numbers per leaf were counted. The length and the width of basal leaflets, middle leaflets (only those with more than 3 leaflets), and top leaflets of each compound leaf were measured with a caliper.

### Data Analysis

Sequences of ITS, *trn*G*-trn*S, and *petL-psbE* were assembled with DNAStar (Gene Codes Cooperation, USA), aligned with Clustal X version 1.81 (igbmc u-strasbg, France; Thompson et al., 1997), and manually adjusted in BioEdit version 7.0.4.1 (Tom Hall, Ibis Therapeutics, USA; Hall, 1999). Indels in the cpDNA dataset were treated as single mutation and coded as substitution (A or T). Diversity and differentiation parameters (within-population diversity, *h*_*S*_; total diversity, *h*_*T*_; differentiation for unordered and ordered alleles, *G*_ST_ and *N*_ST_, respectively) were calculated with HAPLONST (Pons and Petit, 1996). This program was also used to test whether *N*_ST_ is larger than *G*_ST_ (Pons and Petit, 1996). The significance of the difference between *N*_ST_ and *G*_ST_ was assessed with one thousand random permutations following Burban et al. (1999). An analysis of molecular variance (AMOVA) was performed using Arlequin version 3 (CMPG, University of Berne, Switzerland; Excoffier et al., 2005). The 95% CIs for *F*_ST_ values were obtained using 10,000 permutations. Gene flow among populations was calculated by the formula: *N*m = 0.25 × (1 – *G*_ST_)/*G*_ST_ (Wright, 1931).

Spatial genetic structure was analyzed with SAMOVA 1.0 (Dupanloup et al., 2002). The *F*_CT_ index of genetic differentiation among K groups was computed to obtain the best configuration of groups. In this study, the tested K values ranged from 2 to 20 for chloroplast haplotypes and 2–24 for ITS haplotypes, with each simulation starting from 100 random initial conditions and 1,000 permutations. Tajima's D (Tajima, 1989), Fu and Li's F (Fu, 1997) statistics, and mismatch distribution analysis were also calculated using Arlequin version 3 (Excoffier et al., 2005) with 1,000 parametric bootstrap replicates to test for selective neutrality and for evidence of range expansion. Observed and inferred haplotypes were nested according to Posada and Crandall (2001). Network reconstruction and Nested Clade Phylogeographic Analysis (NCPA) were carried out using ANeCA software (University of Vigo, Spain; Panchal, 2007). The closed loops were resolved using the rules and predictions based on coalescence theory (Crandall and Templeton, 1993; Templeton and Sing, 1993). Finally, a list of inference keys for the origin of the nested clades was generated.

Phylogenetic relationships among the haplotypes of the complex and the outgroups were reconstructed using both maximum likelihood (ML) and Bayesian inference (BI). Prior to phylogenetic analyses, sequences were aligned using MAFFT (Osaka University, Japan; Katoh and Standley, 2013). Based on the AIC calculated from the MrModetest 2.3 (Uppsala University, Sweden; Nylander, 2004), the GTR+I was the best-fitting substitution model for both the ML and BI analyses. The ML analysis was performed using RAxML 8.2.11 (Heidelberg Institute for Theoretical Studies, Germany) with 1,000 bootstrap replicates (Stamatakis, 2014). The BI analysis was conducted using Mrbayes 3.2.6 (University of Rochester, USA and Uppsala University, Sweden; Huelsenbeck and Ronquist, 2001). The Markov chain Monte Carlo algorithm was run for 1,000,000 generations with four incrementally heated chains, starting from random trees and sampling every 1,000 generations. The first 25% of the trees were discarded as burn-in and the remaining trees were used to construct a 50% majority-rule consensus tree. Internodes with posterior probabilities > 70% were considered statistically significant. A log-likelihood ratio test (LRT) was first performed using MEGA 10.2.2 (Tokyo Metropolitan University, Japan; Kumar et al., 2018) to estimate divergence times and to test the existence of a constant substitution rate among the cpDNA haplotypes by comparing the log L scores of ML trees with or without a molecular clock enforced, following the test statistic: −2 (log L_clock_ – log L_noclock_), which should be distributed as χ^2^ with (*N* – 2) degrees of freedom (*df*), where *N* is the number of sequences in the tree (Posada and Crandall, 1998). Then, as no fossils are available to calibrate the intergenic spacers substitution rate for genus *Rosa*, branch lengths of the clock-constrained ML tree of cpDNA were transformed into absolute time by assuming the substitution rates of these spacers to be (1.2–1.7) × 10^−9^ substitutions per site per year (s/s/y) (Graur and Li, 2000).

For leaf morphology analysis, the ratio of length to width of a leaflet was calculated to indicate its shape. One-way ANOVA was used to test for statistical differences between the two taxa. Statistical analyses were done using Excel 2007 (Microsoft Corporation, USA).

### Population Prioritization for Conservation

In order to rank each population by its conservation priority, we used Contrib ver.1.02 (Petit et al., 1998) to estimate the contribution of each population to total diversity (*CT*) and to total allelic differentiation (*CrT*). The data of cpDNA haplotypes and their distribution among populations were used as input data for this analysis. Negative values indicate that the diversity or the differentiation of a population is lower than the mean of the whole dataset (Petit et al., 1998; Swatdipong et al., 2009). The higher population of *CT* or *CrT* is, the higher the conservation priority this population has.

## Results

### Leaf Morphology Comparison

The number and size of the leaflets of *R. chinensis* var. *spontanea* and *R. lucidissima* were shown in Table 3. About 56.2% individuals of *R. chinensis* var. *spontanea* has 5 leaflets per compound leaf while 67.9% individuals of *R. lucidissima* has only 3 leaflets. The top leaflets of both taxa are larger than the middle and the basal ones. Averagely, the leaflets at the same position of *R. lucidissima* are longer and narrower than those of *R. chinensis* var. *spontanea*, despite that the difference is not significant. However, the ratio of length to width of all the leaflets of *R. chinensis* var. *spontanea* is significantly less than that of *R. lucidissima*, indicating the leaflets of *R. chinensis* var. *spontanea* are much broader than those of *R. lucidissima*.

**Table 3:** Comparison of compound leaves between *Roa chinensis* var. *spontanea and Rosa lucidissima*.

**Taxa**	**Leaflet number**	**Top leaflet (cm)**			**Middle leaflet (cm)**			**Basal leaflet (cm)**		
		**Length**	**Width**	**Length/width**	**Length**	**Width**	**Length/width**	**Length**	**Width**	**Length/width**
*R.chinensis* var. *spontanea*	4.64 ± 0.65	4.96 ± 0.83	2.62 ± 0.61	1.93 ± 0.17	3.89 ± 0.46	2.04 ± 0.36	1.95 ± 0.23	3.38 ± 0.71	1.85 ± 0.45	1.85 ± 0.16
*R.lucidissima*	3.52 ± 0.72	5.19 ± 0.97	2.30 ± 0.39	2.27 ± 0.29	4.62 ± 0.37	2.08 ± 0.56	2.24 ± 0.12	3.65 ± 0.75	1.73 ± 0.38	2.16 ± 0.25
Significance (P)	0.050	0.549	0.219	0.0015^**^	0.106	0.898	0.035^**^	0.414	0.509	0.006^**^

*Data were Means ± SE*,

** *means significant difference between the two taxa*.

### Haplotypes, Genetic Diversity, and Structure Based on CpDNA Fragments

The aligned sequences of *trn*G-*trn*S and *pet*L-*psb*E of the *R. chinensis* var. *spontanea* and *R. lucidissima* complex (hereafter *R. chinensis* var. spontanea - *R. lucidissima* complex) was 1,359 and 1,213 bp in length, respectively. A total of 25 polymorphic sites and 10 indels were detected in the concatenated dataset. In total, there were 22 haplotypes (H1-H22) and their GenBank accession numbers were shown (Supplementary Table S1). *Rosa chinensis* var. *spontanea* had 9 haplotypes and *R. lucidissima* had 14 haplotypes. The only haplotype shared by both taxa was H3.

The spatial distribution and relative frequencies of the haplotypes are shown in Figure 1A and Table 1. Haplotypes H4, H6, H8, H13, and H14 all were represented by a single extant individual. The haplotype H6 was found in *R. chinensis* var. *spontanea* and the others in *R. lucidissima*. Haplotype H3 (158/330 individuals) was present in 12 out of the 16 *R. chinensis* var. *spontanea* populations and in 2 out of the 9 *R. lucidissima* populations. All the 14 populations with H3 were on the boundary of the Sichuan Basin, such as WuS and YC, etc. east in the Three Gorges Mountain Region (TGMR), NJ and PC north in the southern Qingling-Dabashan Mountains, WX and MX northwest in the Longmenshan Mountains, MB and LB southwest in the Xiaoliangling Mountains. Although not detected in the south edge of Sichuan Basin, H3 existed more south to DJ and WA where the south edge of Sichuan Basin transited to the northeastern Yunnan-Guizhou Plateau. Haplotype H5 was only found in northern populations of *R. chinensis* var. *spontanea* (PW, CX, and NJ). Except for H3 and H5, the other seven haplotypes were all “private” to a single population of *R. chinensis* var. *spontanea*. Among the 14 haplotypes detected in *R. lucidissima*, except for the shared haplotype H3, all others were private ones.

Three populations had three haplotypes. Most individuals in population HJ were haplotype H7, two individuals were haplotype H2 and the rest had haplotype H8. Besides the widespread haplotype H3, population LB also had two more haplotypes. Most individuals of population LZ in southwestern Guangxi were haplotype H12, and the other two individuals belonged to H13 and H14, respectively. Five populations had two haplotypes. Populations BD and FJ in the TGMR, and population NJ in the Qingling-Dabashan Mountains, had another private haplotype (H4, H6, and H5, respectively) besides H3. Most individuals in population AL had haplotype H1, while three individuals were H2. Most individuals in population NC were haplotype H9 and two individuals were H16. The other populations had only one haplotype.

The chloroplast genetic diversity (*h*_T_) of the complex was 0.768 ± 0.085, that of *R. chinensis* var. *spontanea* was 0.596 ± 0.12 and that of *R. lucidissima* was 0.972 ± 0.04. The genetic diversity within the population (*h*_S_) of the complex was 0.133 ± 0.037, that of *chinensis* var. *spontanea* was 0.122 ± 0.048 and that of *R. lucidissima* was 0.154 ± 0.061. The total haplotype diversity (*H*_d_) and the nucleotide (Pi, π) of the *R. chinensis* var. spontanea *- lucidissima* complex and each taxa are shown in Table 1. For the complex, AMOVA revealed that most of the variation (92.29%) is among populations (Supplementary Table S3). In *R. chinensis* var. *spontanea*, the among population component was 89.54%, and in *R. lucidissima*, it was 92.44%. The *N*m calculated for the complex was 0.053, and for *R. chinensis* var. *spontanea* and *R. lucidissima* it was 0.064 and 0.047, respectively, meaning extremely low gene flow among populations. The genetic diversity analysis revealed that genetic differentiation among populations of the complex (*N*_ST_ = 0.93 ± 0.02, *G*_ST_ = 0.83 ± 0.04), *R. chinensis* var. *spontanea* (*N*_ST_ = 0.89 ± 0.06, *G*_ST_ = 0.8 ± 0.08) and *R. lucidissima* (*N*_ST_ = 0.93 ± 0.03, *G*_ST_ = 0.84 ± 0.06) all was high. The comparison of *N*_ST_ and *G*_ST_ indicated a lack of phylogeographic structure either in the complex (*p* = 0.245), *R. chinensis* var. *spontanea* (*p* = 0.152), or in *R. lucidissima* (*p* = 0.999).

The SAMOVA revealed, especially when the two taxa were analyzed separately, a strong structure of genetic variation. For the complex, *F*_CT_ varied irregularly making it impossible detection of the optical K value (Supplementary Figure S1A). For *R. chinensis* var. *spontanea*, the optimal *F*_CT_ was found with *K*=5, indicating populations could be divided into 5 groups. The single population LB of southwestern Sichuan was Group I. Group II included populations CX, NJ, and PW of northern Sichuan. The single population RH in northern Guizhou was Group IV and the single population NC on the southeastern border of Sichuan Basin was Group V. Group III included all the other populations. The optimal *F*_CT_ of *R. lucidissima* was found with K = 4, implying the population could be divided into 4 groups. Group, I included population HJ, LZ, and WS; population DJ, WA, and YY formed Group II; the single population PS was Group III; and populations AL and SB formed Group IV.

In the population neutrality test applied to either the complex or each of the two taxa, the D value did not deviate significantly from zero (Table 4). The test of mismatch distribution of the number of pairwise nucleotide differences for cpDNA haplotypes showed that the observed value departed from the expectation with the multimodal distribution (Supplementary Figure S2A), indicating a lack of recent range expansion within each taxon and the whole complex.

**Table 4:** The results of Tajima's test and Fu and Li's test of *R. chinensis* var. *spontanea-lucidissima* complex and each taxon.

**Taxa**	**Tajima's test**	**Fu and Li's test**	
	**D**	D*	F*
*R. chinensis* var. *spontanea -lucidissima* complex	0.86016 (*P* > 0.10)	1.90954 (*p* < 0.02)	1.77600 (*p* < 0.05)
*R. chinensis* var. *spontanea*	1.40005 (*P* > 0.10)	1.55297 (*p* < 0.02)	1.79885 (*p* < 0.05)
*R. lucidissima*	0.90757 (*P* > 0.10)	1.74811 (*p* < 0.02)	1.70509 (0.10 > *P* > 0.05)

### Ribosome Haplotypes, Genetic Diversity, and Structure Based on ITS

The aligned 422 ITS sequences had 642 nucleotide positions of which 26 were polymorphic. In total, 21 ITS haplotypes (ribotypes) were identified and sequence variation for ribotypes can be seen in Supplementary Table S2. There were 13 and 11 ribotypes specific to *R. chinensis* var. *spontanea* and *R. lucidissima*, respectively, while rH2, rH3, and rH5 were shared by the two taxa. The spatial distribution and relative frequencies of the ribotypes are presented in Table 1 and Figure 1B.

The ribotype rH3 (206 of 422 sequences) predominated in the complex, especially in *R. chinensis* var. *spontanea*. Ribotype rH3 was present in almost all populations of *R. chinensis* var. *spontanea* and in two populations (PS and DJ) of *R. lucidissima*. These populations were around the Sichuan Basin. The second most popular ribotype was rH2, present in the majority of the populations of *R. lucidissima*, but in only one population of *R. chinensis* var. *spontanea* population (RH). Ribotype rH5 was found in populations of both taxa at the conjunction of Sichuan Basin and Yunnan-Guizhou Plateau, namely in populations NC, PS, YY, and DJ. Ribotype rH12 was found in *R. chinensis* var. *spontanea* populations of MB, RS, and PW on the western border of the Sichuan Basin. rH20 was in ZG and YC of *R. chinensis* var. *spontanea*, while rH13 was found in *R. lucidissima* populations PS and WA. The other ribotypes were all private. Among them, ribotypes rH4, rH10, and rH19 were represented by a single individual each.

The ITS genetic diversity (*h*_T_) of the complex was 0.726 ± 0.075, that of *R. chinensis* var. *spontanea* was 0.464 ± 0.107 and that of *R. lucidissima* was 0.907 ± 0.031. The genetic diversity within the population (*h*_S_) of the complex was 0.344 ± 0.051, that of *chinensis* var. *spontanea* was 0.295 ± 0.07 and that of *R. lucidissima* was 0.431 ± 0.064. The total haplotype diversity (*H*_d_) and the nucleotide (Pi, π) of the complex and each taxa based on ITS are shown in Table 1. For the complex, AMOVA revealed over half of the variation (58.90%) is among populations (Supplementary Table S3). In *R. chinensis* var. *spontanea*, the among population component was 55.72%, and in *R. lucidissima*, it was 40.22%. The *N*m calculated for the complex was 0.225, and for *R. chinensis* var. *spontanea* and *R. lucidissima* it was 0.434 and 0.226, respectively. The genetic differentiation of the whole complex (*G*_ST_ = 0.53 ± 0.06, *N*_ST_ = 0.64 ± 0.08) and *R. lucidissima* (*G*_ST_ = 0.53 ± 0.08, *N*_ST_ = 0.52 ± 0.13) was higher than *R. chinensis* var. *spontanea* (*G*_ST_ = 0.37 ± 0.07, *N*_ST_ = 0.54 ± 0.15).

In SAMOVA, the optimal *F*_CT_ value for *R. chinensis* var. *spontanea - R. lucidissima* complex was found for K = 3 (Supplementary Figure S1B), with Group I comprising almost all populations around the Sichuan Basin; Group II including populations of Yunnan-Guizhou Plateau and adjacent areas; and Group III comprising population LB in southwestern Sichuan and WS in southeastern Yunnan. For *R. chinensis* var. *spontanea*, SAMOVA identified two groups: Group I with only population LB, and Group II with all the remained populations. For *R. lucidissima*, *F*_CT_ was changing irregularly with an increase in K value, indicating no group structure.

While Tajima's D values were not significant, D^*^ and F^*^ in Fu and Li's test significantly deviated from zero (Table 4), indicating a bottleneck effect and possible balancing selection, and the test of mismatch distribution revealed departure from the expectation with bimodal distribution (Supplementary Figure S2B), indicating that populations in the entire geographical region lack of recent range expansion. This was also found both in the whole complex and for the two taxa analyzed separately.

### Genetic Relationship of Populations, Phylogenetic and Nested Clade Analysis

**Figure 3:**
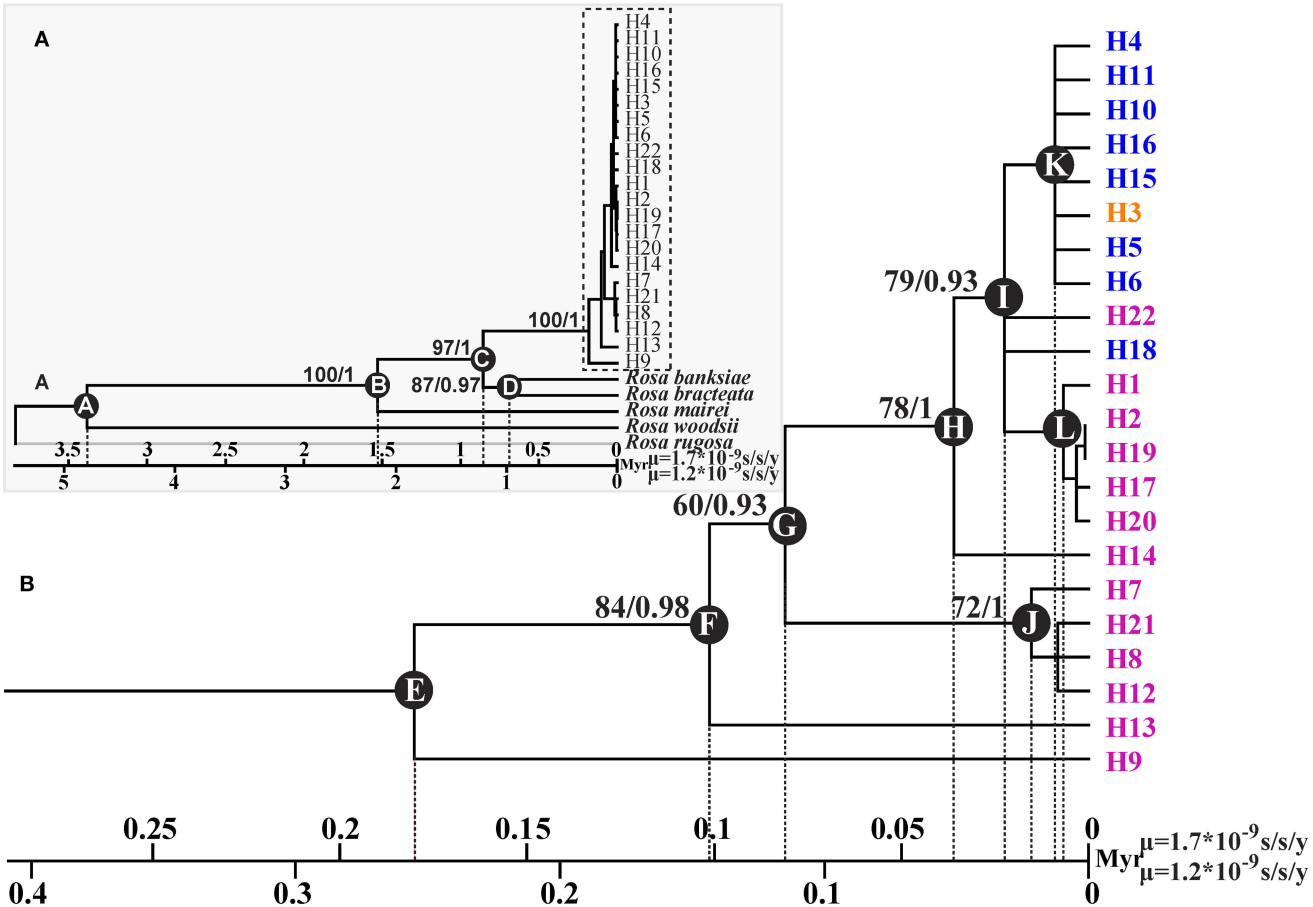
Phylogenetic tree and divergence time estimates of cpDNA haplotypes of *R. chinensis* var. *spontanea*- *Rosa lucidissima* complex. Part **A** showed the phylogenetic relationship and divergence time between the complex and the outgroup species, while part **B** showed those within the complex. *Rosa lucidissima* haplotypes are in pink, *R. chinensis* var. *spontanea* ones in blue, and the shared ones in orange. Bootstrap values (%) (ML) and the posterior probability values (BI) are indicated above the branches. The estimated divergence time among the outgroup species, between the complex and outgroups, and for the complex clades are indicated at nodes: A (3.38–4.79 Mya), B (1.53–2.16 Mya), C (0.86–1.21 Mya), D (0.68–0.95 Mya), E (0.18–0.25 Mya), F (0.1–0.14 Mya), G (0.08–0.11 Mya), H (0.036–0.05 Mya), I (0.02–0.03 Mya), J (0.01–0.02 Mya), K (0.008–0.01 Mya), L (0.006–0.008 Mya).

GenBank accession numbers from OL774542 to OL774546 were for the *trn*G-*trn*S sequences of *R. banksiae, R. bracteata, R. mairei, R rugosa*, and *R. woodsii*, respectively, and those from OL774562 to OL774566 were for their *pet*L-*psb*E sequences, respectively. The ML and BI applied to cpDNA data produced trees with identical topology (Figure 3). The 22 haplotypes identified in *R. chinensis* var. *spontanea - R. lucidissima* complex formed one clade that diverged from other congeneric species at about 1.21–0.86 Mya (node C). The majority of the haplotypes of *R. lucidissima* evolved much earlier than those of *R. chinensis var. spontanea*. Within *R. lucidissima*, haplotypes H9 and H13 are the most ancestral that diverged about 0.25–0.18 and 0.14–0.1 Mya (node E and F, respectively). The group of haplotypes H7, H21, and H8 diverged the next at about 0.08–0.11 Mya (node G) followed by haplotype H14 at about 0.036–0.051 Mya (node H). An ancestor that evolved at that time (node H), gave rise to a large number of haplotypes of both *R. lucidissim* and *R. chinensis* var. *spontanea*. Within this clade, the ancestral haplotype H18 of *R. chinensis* var. *spontanea* diverged from *R. lucidissim* haplotypes about 0.022–0.031 Mya (node I), and a group of *R. lucidissim* haplotypes H1, H2, H17, H19, H20 diverged about 0.006–0.008 Mya (node L). In *R. chinensis* var. *spontanea*, except for the most ancestral haplotype H18, other haplotypes diverged very recently at about 0.0086–0.012 Mya (node K).

**Figure 4:**
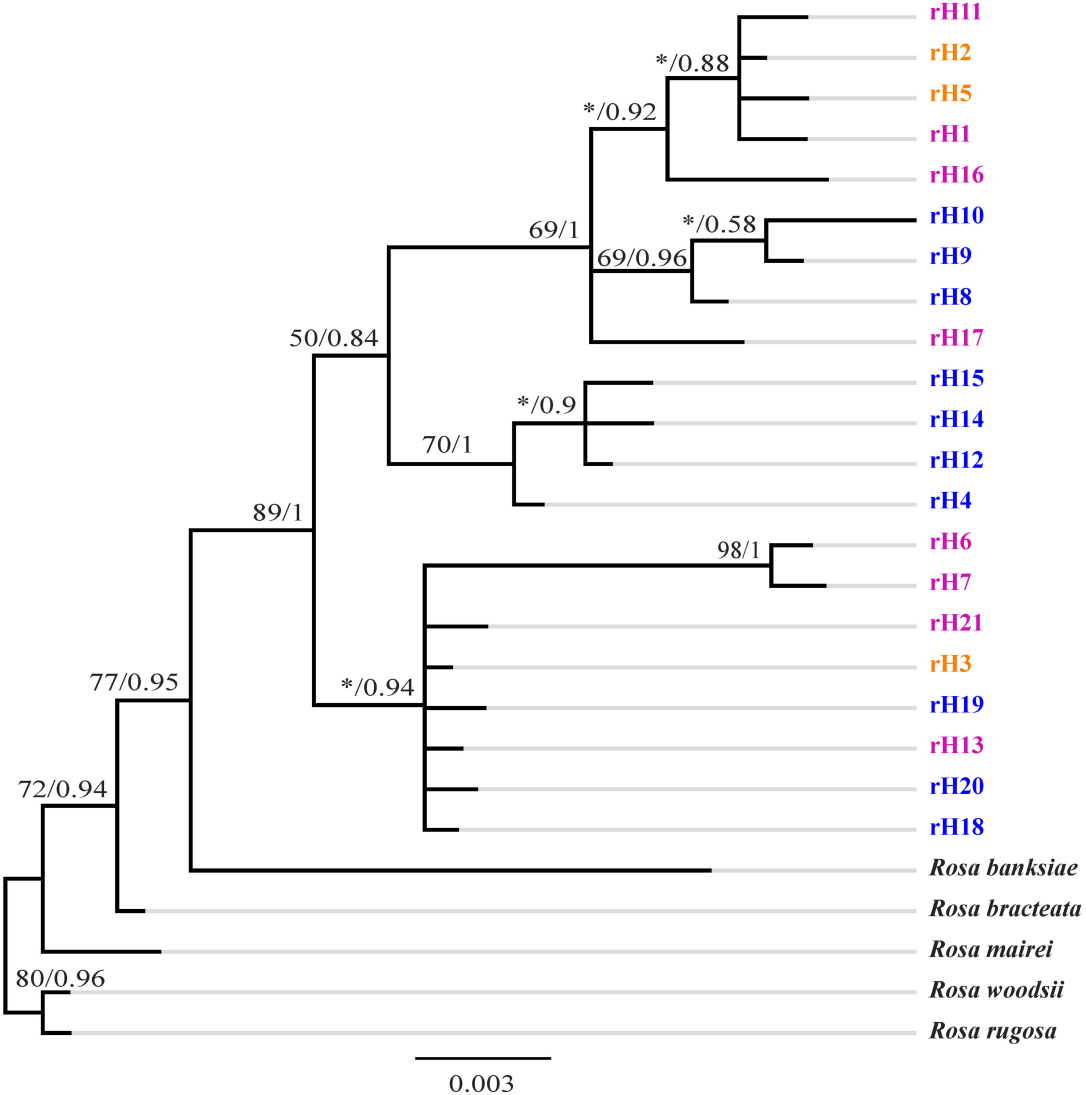
Phylogenetic tree of ribotypes of *R. chinensis* var. *spontanea* and *R. lucidissima* complex. Bootstrap values (%) (ML) and the posterior probability values (BI) are indicated above the branches. *R. lucidissima* haplotypes are in pink, *R. chinensis* var. *spontanea* ones in blue, and the shared ones in orange. *Means the bootstrap value is less than 50%.

GenBank accession numbers from OL719054 to OL719058 were ITS sequences of *R. bracteata, R. banksiae, R rugosa, R. woodsii*, and *R. mairei*, respectively. The ITS ribotypes identified in *R. chinensis* var. *spontanea*- *R. lucidissima* complex also formed with high support of a separate clade in the tree (Figure 4). This clade had two distinct subclades. The first subclade included ribotypes rH18, rH19, rH20 of *R. chinensis* var. *spontanea* from the TGMR areas, the widely distributed rH3 shared by both *R. chinensis* var. *spontanea* and *R. lucidissima* around the Sichuan Basin, rH21 of *R. lucidissima* in PS and YY at the southeastern Sichuan basin near the Wulingshan Mountains, and rH6 and rH7 of *R. lucidissima* in HJ in northern Guangxi. The second one was further divided into two paraphyletic branches. The first contained rH4, rH14, rH12, and rH15 of *R. chinensis* var. *spontanea* from the northern and northwestern border of the Sichuan Basin. The second branch included rH1, rH11, and rH16 of *R. lucidissima* from AL, LZ, and SB, rH2 and rH5 shared by both taxa from NC, DJ, YY, and SB in the conjunction of Yunnan-Guizhou Plateau and southeastern Sichuan Basin, rH8 and rH9 of *R. chinensis* var. *spontanea* from LB in southwestern Sichuan and rH17 of *R. lucidissima* from WS in southeastern Yunnan.

**Figure 5:**
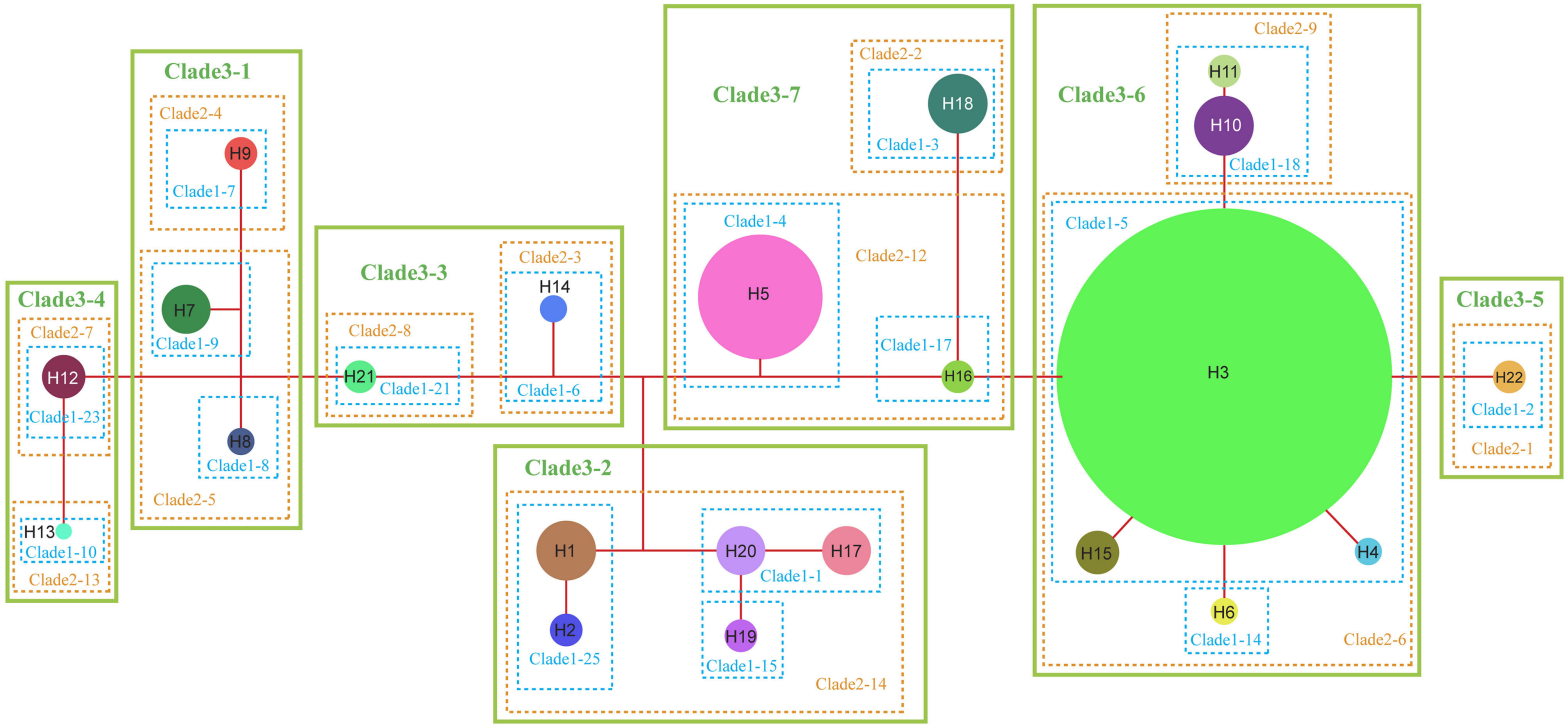
Nested cladogram of the chloroplast haplotypes (H1–H22) of *R. chinensis* var. *spontanea* and *R. lucidissima* complex. Circles with numbers denote haplotypes and their sizes are proportional to the observed frequencies of the haplotypes. Dots represent putative haplotypes. Each branch represents one mutation.

The NCPA applied to the chloroplast haplotype data produced a network of seven main clades (Figure 5). For only a few of them, the inferences of historical events that could shape their present-day spatial distribution can be made, with no overall conclusive inference for the complex evolution (Supplementary Table S4). Both the interior-clade 1-1 is formed by H20 (SB) and H17 (PS), and the subclade 2-12 is formed by interior-clade 1-4 (H5, NJ, and PW) and interior-clade 1-17 (H16, NC) could result from “allopatric fragmentation”. The clade 3-7 formed by subclade 2-12 and subclade 2-2 (H18, RH) could result from “allopatric fragmentation”, too. The interior-clade 1-5 including widely distributed H3, H4 from BD, H6 from FJ and H15 from NC could result from “restricted gene flow with isolation by distance”. The NCPA for ribotypes also produced a network of seven main clades (Supplementary Figure S3). At the complex level, no conclusive inference could be made for the historical events that shaped the present-day ribotype spatial distribution (Supplementary Table S4). Only subclade 1-3 including rH1 and rH2 from AL, rH11 from LZ was inferred as a result of “restricted gene flow/dispersal but with some long-distance dispersal over intermediate areas not occupied by the species; or past gene flow followed by extinction of intermediate populations”.

### Contribution of the Extant Populations to CpDNA Variation

**Figure 6:**
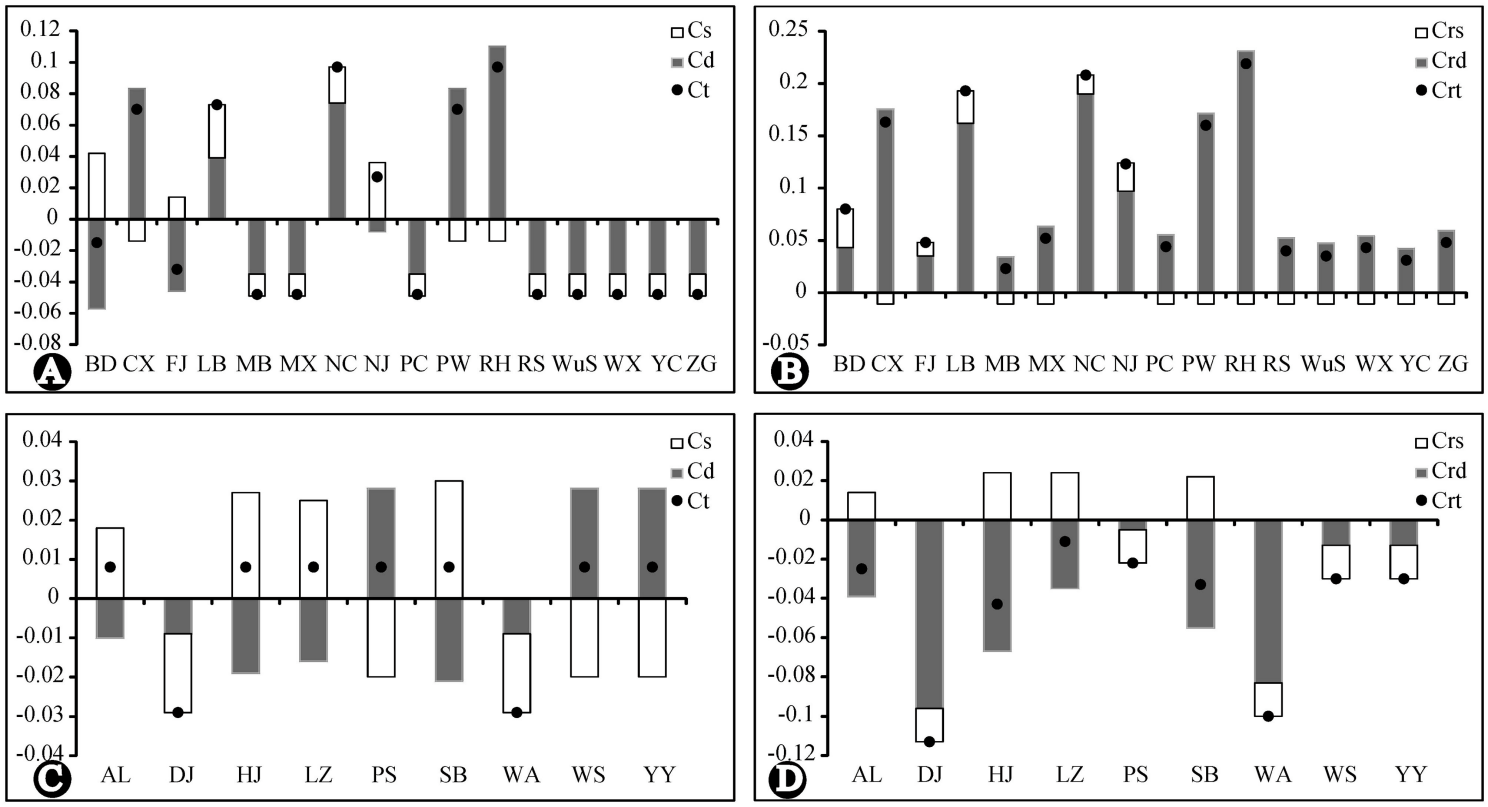
Contribution of each population to the genetic and allelic diversity of *R. chinensis* var. *spontanea* and *R. lucidissima* complex. *Cs*, the contribution of genetic diversity. *Cd*, the contribution of genetic differentiation. *CT*, the contribution of total diversity. *Crs*, the contribution of allelic diversity. *Crd*, the contribution of allelic differentiation. *CrR*, the contribution of total allelic richness. **(A,B)**: *R. chinensis var.spontanea*. **(C,D)**: *R.lucidisssima*.

The results of the analysis of population contribution to genetic diversity by Contrib are shown in Figure 6. The population RH, NC, PW, LB, CX, and NJ made the highest contribution to the total cpDNA genetic diversity and allelic diversity of *R. chinensis* var. *spontanea*. Among them, population RH, PW and CX contributed due to their high genetic differentiation (Cd) from others, even though they had low genetic diversity (Cs), while NC and LB had both high genetic differentiation and high genetic diversity. Contributions of the population of *R. lucidissima* (except for WA and DJ having low contribution) to both genetic diversity and allelic diversity was similarly high.

## Discussion

### Species Boundary Between *R. chinensis* var. *spontanea* and *R. lucidissima*

Wild rose species are characterized by extensive morphological variation, which has resulted in a notoriously complex taxonomy (Wissemann, 2003; Joly et al., 2006), e.g., *R. caninae* complex (De Cock et al., 2008), *R. soulieana* (Jian et al., 2015), *R. sericea* complex (Gao et al., 2015, 2019) and *R. chinensis* var. *spontanea* and *R. lucidissima* complex. *Rosa chinensis* var. *spontanea* and *R. lucidissima* are very similar morphologically to each other (Henry, 1902; Léveillé, 1911; Ku, 1985; Ku and Robertson, 2003; Rix, 2005), and taxonomic circumscription between the two taxa is confusing both in herbarium sheets and in the field. Studies focusing on natural population are critical for understanding not only *Rosa* sections *Chinenses* and *Synstylae* but even the whole genus since many closely related species resulted from a fast diversification, incomplete lineage sorting or reticulate evolution (Zhu et al., 2015). In our study, *R. chinensis* var. *spontanea* and *R. lucidissma* shared one cpDNA haplotype and three ribotypes, mainly in a transitional area between southeastern Sichuan Basin and Yunnan-Guizhou Plateau, where populations of the two taxa meet. Secondly, although both taxa have many haplotypes limited to a small region or occurring in a single population, spatial distribution of the haplotypes roughly corresponds to the taxa boundaries based on morphology. Thirdly, populations of the whole complex were divided into two main clades based on the genetic distance among populations based on both ITS and cpDNA (Supplementary Figure S4). Fourthly, the ecological niches of *R. chinensis* var. *spontanea* and *R. lucidissma* only overlap at the transition zone between Yunnan-Guizhou Plateau and southeastern border of Sichuan Basin. So, we suggest *R. chinensis* var. *spontanea* and *R. lucidissma* be treated as independent taxa, according to morpho-geographical species concept (Mitaka, 2003). The northern populations of the complex, mainly those around and within the Sichuan Basin were *R. chinensis* var. *spontanea*, which has broader leaflets and paler full-blooming flowers in general, and those of the southern populations, mainly those distribute in Guangxi, Yunnan, Middle and Southern Guizhou, with narrower leaflets and darker flowers, were *R. lucidissma* (Supplementary Figure S5). The southeastern Sichuan Basin and the northeastern Guizhou are the contact or the hybridization zone of the two taxa. A large panel of SNPs at a genome-level should be necessary for a more precisely delimiting of both taxa in these transitional areas.

### Genetic Differentiation Within and Between *R. chinensis* var. *spontanea* and *R. lucidissma*

Southwestern China was weakly but widely affected by the Quaternary Glacials (Committee of Chinese Academy of Sciences for Physical Geography of China, 1984; Liu, 1991). The mountain boundaries between the Second and Third ladders in China were not only the obstacles that retarded gene flow between both sides of the mountains but also the corridors of plant dispersal (Hoorn et al., 2013). The Nanling Mountains (Wang et al., 2009; Kou et al., 2016), Daloushan Mountains (Ma et al., 2015), Wushan and Dabashan Mountains (Liu et al., 2013) all have played such a role in the Sino-Japanese Flora (reviewed by Ye et al., 2017b and references therein). The subtropical flora of China has been traditionally thought to be originated from southwestern China and expanded eastward through such mountain corridors (Wang, 1992a,b). As parts of this flora, *R. chinensis* var. *spontanea – R. lucidissima* complex originated and migrated like this. Haplotypes of *R. lucidissima* diverged from other roses at about 0.86–1.21 Mya presumably in the Günz glacial substage of the Quaternary Oscillation mainly in mountain areas of southeastern Yunnan-Guizhou Plateau and adjacent regions. During the third glacial advance in the Quaternary Oscillation (0.37–0.24 Mya), the ancestors of the southern haplotypes apparently survived on the Jiuwan Mountain in Huanjiang (HJ), where there exist many relic plant species of the Quaternary Glacials (Compilation Committee of Local Chronicles of Guangxi Zhuang Autonomous Region, 1994). The ancestral haplotypes of the middle ones dispersed north to the Daloushan and then east to the Wulingshan Mountains at the northeastern edge of Yunnan-Guizhou Plaeteau and southeastern of Sichuan Basin, where the ancestor of *R. chinensis* var. *spontanea* evolved from *R. lucidissima*, and then both taxa survived the Last Glacial Maximum (LGM) there. So, the Daloushan and Wulingshan Mountains were the complex's refugia in LGM. With the ending of the Quaternary glacials and the coming of the postglacial period, the ancestral haplotypes of the haplotypes in the refugia dispersed northeast along the Wushan Mountains to the Three Gorges Mountains Region and dispersed north along the Ridge and Valleys of the east Sichuan to the Dabashan Mountains, from where the most widely distributed haplotypes (H3) originated and then widely distributed the mountains around the Basin. The southern haplotypes and northern ones within the complex kept being isolated by the environment (IBE) due to the combination of altitude, climate and soil (Bai et al., 2016; Ye et al., 2017a). Then they differentiated further and evolved into the southern *R. lucidissima* and the northern *R. chinensis* var. *spontanea*.

Within *R. chinensis* var. *spontanea*, almost all haplotypes were very recently diverged at about 0.0086–0.012 Mya. For *R. lucidissima*, most haplotypes originated during or even later than this period. On the other hand, almost all cpDNA haplotypes were private to some single population. The reason for this recent and fast diversification of haplotypes might firstly be due to the wide and complex geographical range covering a mosaic of plateaus, mountains, basins, and gorges which could act as geographic barriers of gene flow and thus provided ample opportunity for isolation, drift and mutation (Wang et al., 2009; Ma et al., 2015). Secondly, hybridization between the two taxa, or gene introgression from other rose species could be another reason. It is well known that inter-species hybridization is common in genus *Rosa* (Ritz et al., 2005; Joly and Bruneau, 2006; Schanzer and Kutlunina, 2010; Ritz and Wissemann, 2011; Kellner et al., 2012; Fougère-Danezan et al., 2014). Populations in the contact areas of the two taxa showed richer ribotypes in this study, and we found that populations with higher *H*_d_ value often have individuals with more or less phenotypic traits significantly alike other sympatric rose, such as *R. banksiae* f. *normalis and R. rubus* with same blooming period.

### Population History and Refugia of *R. chinensis* var. *spontanea* and *R. lucidissma*

Our demographic analyses indicated that there was no range expansion of either *R. chinensis* var. *spontanea* or *R. lucidissma*, which could be due to their narrow habitat requirements. Both taxa are highly heliophilous and readily colonize deforested locations but can not compete with the evergreen forest vegetation covering the Sichuan Basin and East China at lower altitudes (Yu et al., 2000; Harrison et al., 2001).

Refugial populations are expected to have higher genetic diversity and longer demographic history than populations that evolved later (Tribsch and Schönswetter, 2003; Lowe et al., 2005; Provan and Bennett, 2008). The Yunnan-Guizhou Plateau and its adjacent regions have long been believed to be an important center of origin for the East Asiatic flora (Wang, 1992a,b) and a refugial area for many species in China (Ying, 2001). Populations of *R. lucidissima* in the southeastern Yunnan-Guizhou Plateau and its adjacent regions, especially populations HJ and LZ had much higher genetic diversity and harbored much more ancient haplotypes (both cpDNA and ITS) than in the other parts of the taxa range, supporting the above hypothesis. In *R. chinensis* var. *spontanea*, population BD, FJ, NJ, LB, and NC all had high genetic diversity, but only RH within Daloushan in the northeastern Yunnan-Guizhou Plateau adjacent to the Sichuan Basin harbored the ancient haplotype H18. Thus, these areas could be possible refugia for *R. chinensis* var. *spontanea*. The population LB in the southwestern edge of Sichuan Basin (Xiaoxiangling Mountains), and NC in southeastern Sichuan Basin having high diversity but young haplotypes, indicated that these areas might be the contact zone or areas of high immigration from diverse sources of *R. chinensis* var. *spontanea*- *R. lucidissim* complex because of their consistent and favorable local conditions.

### The Genetic Diversity and Genetic Structure of the Complex

Despite with a relatively short evolutionary history (1.21–0.86 Mya), *R. chinensis* var. *spontanea - R. lucidissima* had a relatively high level of chloroplast genetic diversity (h_T_, 0.768), comparable to that of *R. praelucens*, endemic to Shangrila of Yunnan Plateau (0.720, Jian et al., 2018), and the widely distributed *R. omeiensis - R. sericea* complex (0.790, Gao et al., 2019), and even much higher than that of another widely distributed wild rose, *R. soulieana* (0.316, Jian et al., 2015). The complex is also characterized by very high genetic differentiation among populations and extremely low genetic variation within populations (*G*_ST_ = 0.83). This extent and structure of genetic diversity appears to be a result of, on one hand, a wide geographical range, covering a mosaic of plateaus, mountains, basins, and gorges in Southwest China, and, on the other hand, numerous geographic barriers within this range limiting seed dispersal and promoting genetic drift. The comprising complex plants are long-living woody shrubs with hermaphroditic flowers pollinated mainly by moths, bees, and other small insects. The migration distance of these insects is limited to several kilometers (Rands and Whitney, 2011). The seed dispersal, unlike many other species, is by gravity and small mammals. In such rose species as *R. canina* (Herrera, 1984), *R. arvensis* (Vander Mijnsbrugge et al., 2010), *R. multiflora* (Ghosh, 2009), and *R. soulieana* (Jian et al., 2015), the hips are red, often <1 cm in diameter and persist on the plants for a long time and therefore often dispersed by birds. In contrast, the hips of *R. chinensis* var. *spontanea* and *R. lucidissma* are greenish yellow and usually about 2–3 cm in diameter. The hips easily fall off the plants as soon as or even before they ripe. The hips only fall around the mother plants and the achenes (seeds) are dispersed mainly by the vertebrates not the birds, and thus within a rather limited distance. Also, because it is mainly distributed in the middle and lower parts of the mountains and along the valleys, the huge mountains act as the barriers to the pollen and seeds dispersal of *R. chinensis* var. *spontanea* and *R. lucidissima*. All these factors may limit inter-population gene flow and contribute to isolation and genetic differentiation of populations.

However, compared to that of cpDNA, genetic diversity based on nuclear DNA (ITS) within the populations (h_s_) was relatively higher, and resulting in less percent of genetic variation existing among the populations and a higher gene flow among populations. These discordant between cpDNA and ITS may result from different dispersal vectors for seeds and pollen, as gene flows due to pollination might be easier and further than those due to seeds dispersal. And also, hybridization within the complex might decrease the genetic differentiation among populations.

### Conservation Implications

Wild plant resources provide infinite potential for superior breeding to improve the genetic diversity of cultivated plants (Long et al., 2018; Wang et al., 2020). Rose is the world's most important ornamental plant, with economic, cultural, and symbolic value (Gudin, 1999; Hibrand Saint-Oyant et al., 2018; Raymond et al., 2018). It is known that 8 to 15 species contributed to the modern rose cultivars (Leus et al., 2018), and unfortunately, this important genetic pool of modern roses is decaying due to intensive breeding (Matsumoto et al., 1998; Scariot et al., 2006; Leus et al., 2018). Wild rose species germplasm is necessary for resistance to introgression (Young et al., 2019), flower color breeding (Zlesak, 2015), and introduction of specific traits (Leus et al., 2018), and therefore it is necessary to protect *R. chinensis* var. *spontanea*-*R. lucidissima* complex.

During our 2 years of outdoor investigation, we did not find any individuals in Yanhe and Xingren in Guizhou, Lingchuan, Fushui, and Cangwu in Guangxi, where there once had specimens records and local people used to dig the roots of *R. lucidissima* to cure stomach diseases. Local people near several other northern populations told us some companies collected old plants of *R. chinensis* var. *spontanea* as the tree rose rootstocks. Also, populations in the dense forest usually had very slim and weak individuals, which were gradually replaced by other plants. So the endangered status of the whole complex might be the result of the over-exploitation of its medical and ornamental value or reforestation of many originally open habitats. We recommend, based on the results of genetic analysis and the contribution of each population, focusing conservation efforts on the populations RH, NC, PW, LB, CX, and NJ of *R. chinenses* var. *spontanea* having the highest contribution to the total cpDNA genetic diversity and allelic richness. Also, populations BD, FJ, RS, WuS, and YC having private haplo- and ribotypes must be given priority in conservation planning and implementation. In *R. lucidissima*, all populations (except for WA and DJ) have high conservation value according to their genetic contribution and haplotype distribution.

The most appropriate and easy to implement an approach for the conservation of these populations *in situ* are plant micro-reserves (Laguna et al., 2004) allowing legal protection, long-term monitoring, and necessary management actions (Volis, 2016). In addition, living collections preserving the existing species' genetic diversity or special phenotypes in a form of seed orchards or quasi *in situ* collections should be established. Given a low number of the extant populations and the high value of this gene pool for breeding, germplasm nurseries such as, for example, those created in Belgium for autochthonous populations of woody species of Flanders (Vander Mijnsbrugge, 2014), seem to be the especially suited for *Rosa ex situ* approach.

## Data Availability Statement

The datasets presented in this study can be found in online repositories. The names of the repository/repositories and accession number(s) can be found in the article/Supplementary Material.

## Author Contributions

HYJ conceived and designed the article and wrote the manuscript. LZ, HJY, XQQ, and TZ performed the experiment. HYJ, LZ, HZ, and QGW collected the plant materials. HYJ, CLM, and NNZ analyzed the data. All authors contributed to the article and approved the submitted version.

## Funding

This work was financially supported by the National Key R&D Program of China (2019YFD1000400) and the National Natural Science Foundation of China (31760087), the Major Program for Science and Technology of Yunnan Province (202102AE090052 and 202002AA100007).

## Conflict of Interest

The authors declare that the research was conducted in the absence of any commercial or financial relationships that could be construed as a potential conflict of interest.

## Publisher's Note

All claims expressed in this article are solely those of the authors and do not necessarily represent those of their affiliated organizations, or those of the publisher, the editors and the reviewers. Any product that may be evaluated in this article, or claim that may be made by its manufacturer, is not guaranteed or endorsed by the publisher.
