# Supplementary material for: Phylogeography and Population Genetics of Rosa chinensis var. spontanea and R. lucidissima Complex, the Important Ancestor of Modern Roses
Source: Front Plant Sci. 2022 May 20;13:851396. doi: 10.3389/fpls.2022.851396 (PMC9163990; doi:10.3389/fpls.2022.851396)
Supplement: Supplementary file 1 [file Data_Sheet_1.PDF]

Supplementary Materials

1.Supplementary Figures

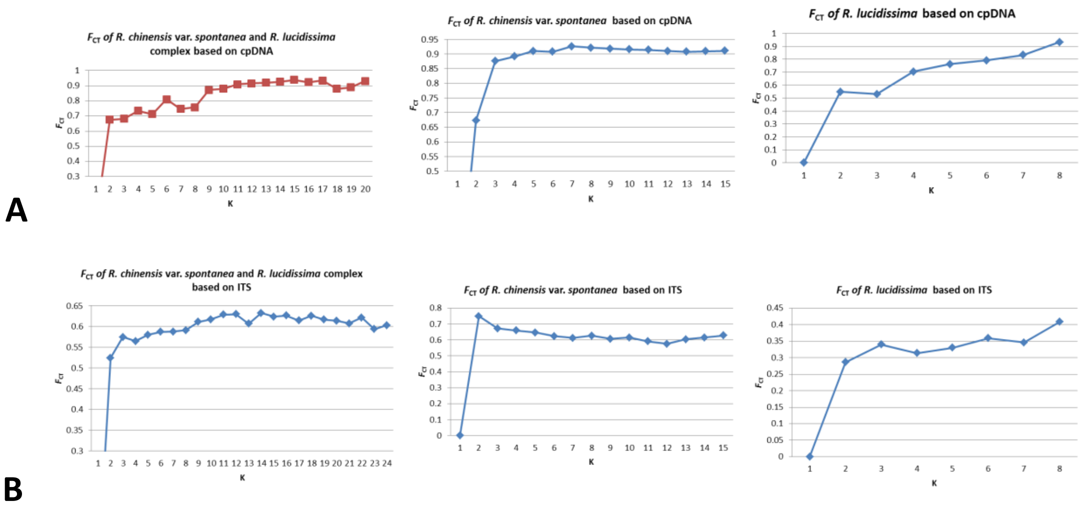

Figure S1. Plots of values of differentiation among groups of populations of *R. chinensis* var. *spontanea* and *R. lucidissima* complex obtained from SAMOVA as a function of the user defined number of groups (K). (A) bsed on cpDNA, (B) based on ITS

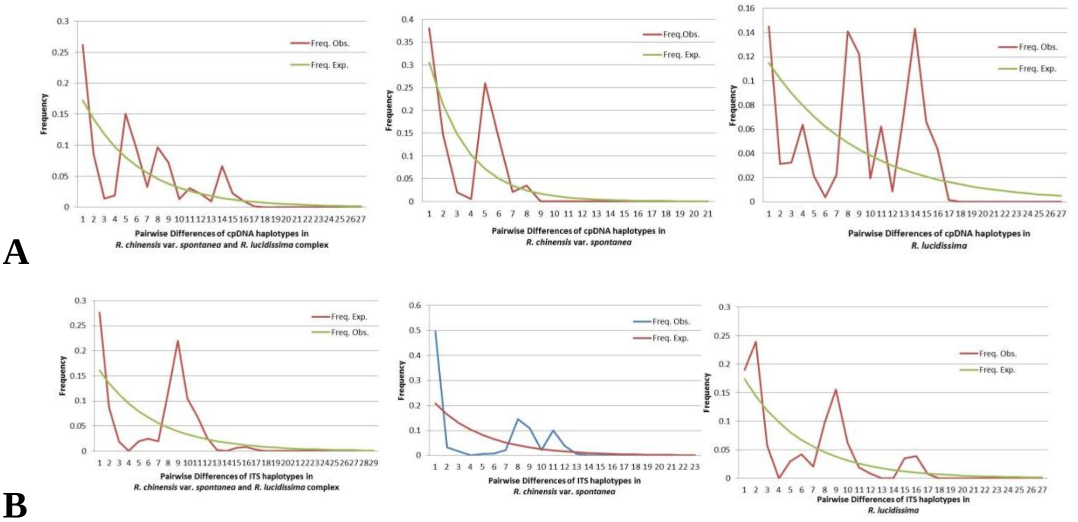

Figure S2. Distribution of the number of pairwise nucleotide differences in *R. chinensis* - *R. lucidissima* complex under a model of sudden (stepwise) population expansion. (A) for cpDNA haplotypes, (B) for ITS ribotypes

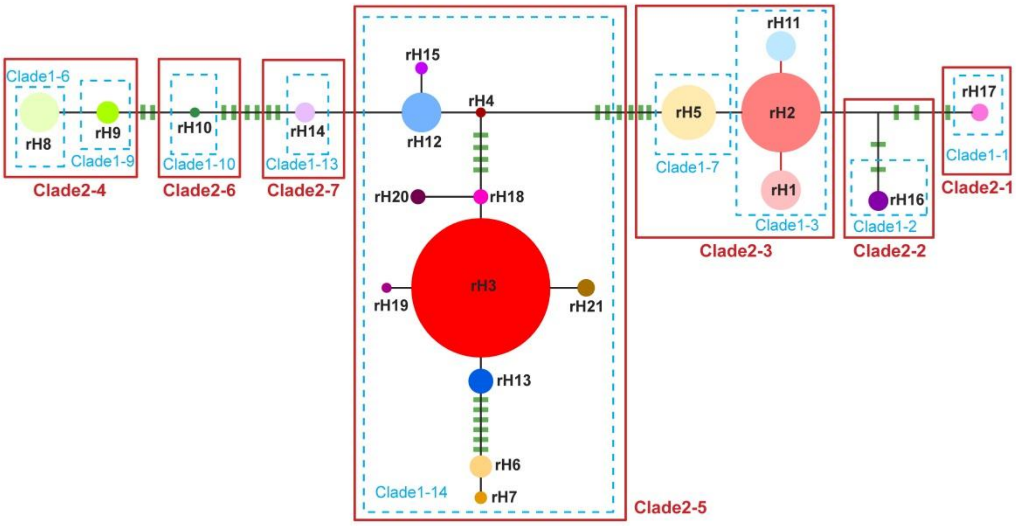

Figure S3. Nested cladogram of the chloroplast haplotypes (rH1–rH21) *R. chinensis* var. *spontanea* and *R. lucidissima* complex. Circles with numbers denote haplotypes and the size of the circles are proportional to the observed frequencies of the haplotypes. Dots represent putative haplotypes. Each branch represents one mutation

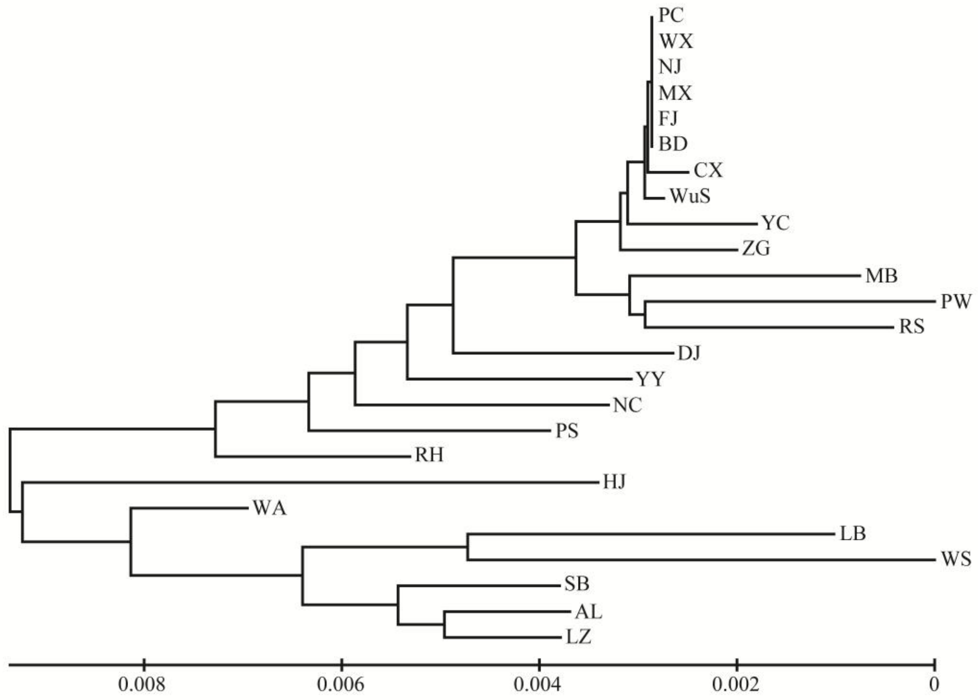

A

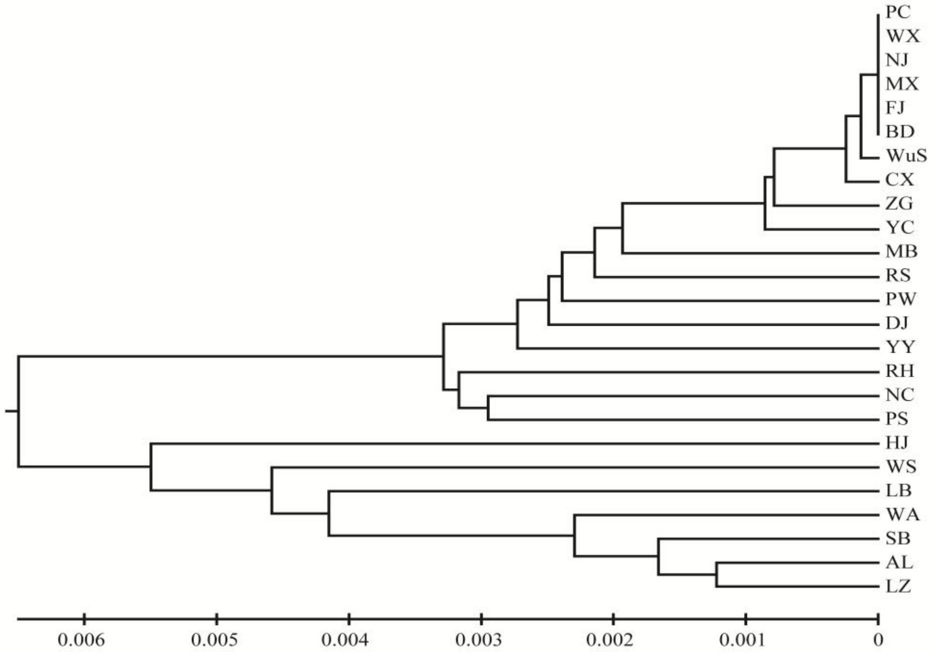
PC
WX
NJ
MX
FJ
BD
WuS
CX
ZG
YC
MB
RS
PW
DJ
YY
RH
NC
PS
HJ
WS
LB
WA
SB
AL
LZ
0.006
0.005
0.004
0.003
0.002
0.001
0

B

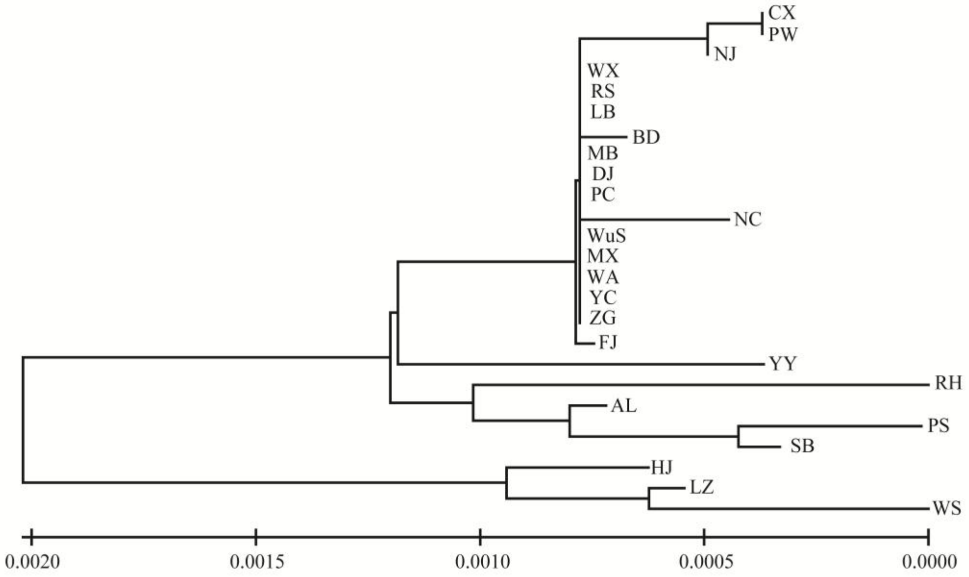
CX
PW
NJ
WX
RS
LB
BD
MB
DJ
PC
NC
WuS
MX
WA
YC
ZG
FJ
YY
RH
AL
PS
SB
HJ
LZ
WS
0.0020
0.0015
0.0010
0.0005
0.0000

C

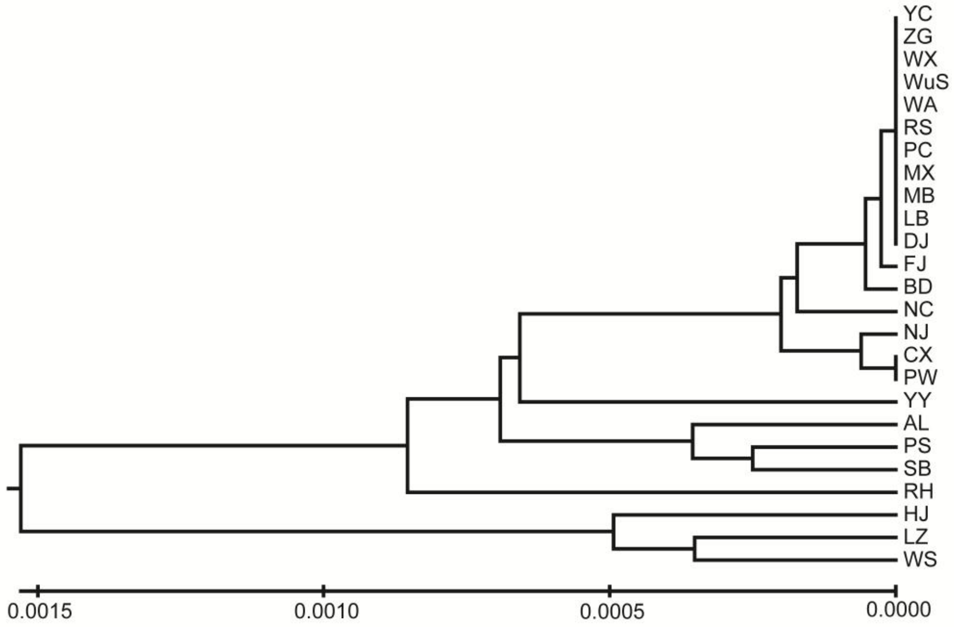

D

Figure S4. Genetic relationship of populations based on ITS and cpDNA of the species complex. (A) NJ tree based on ITS, (B) UPGMA tree baed on ITS, (C) NJ tree based on cpDNA, (D) UPGMA tree based on cpDNA

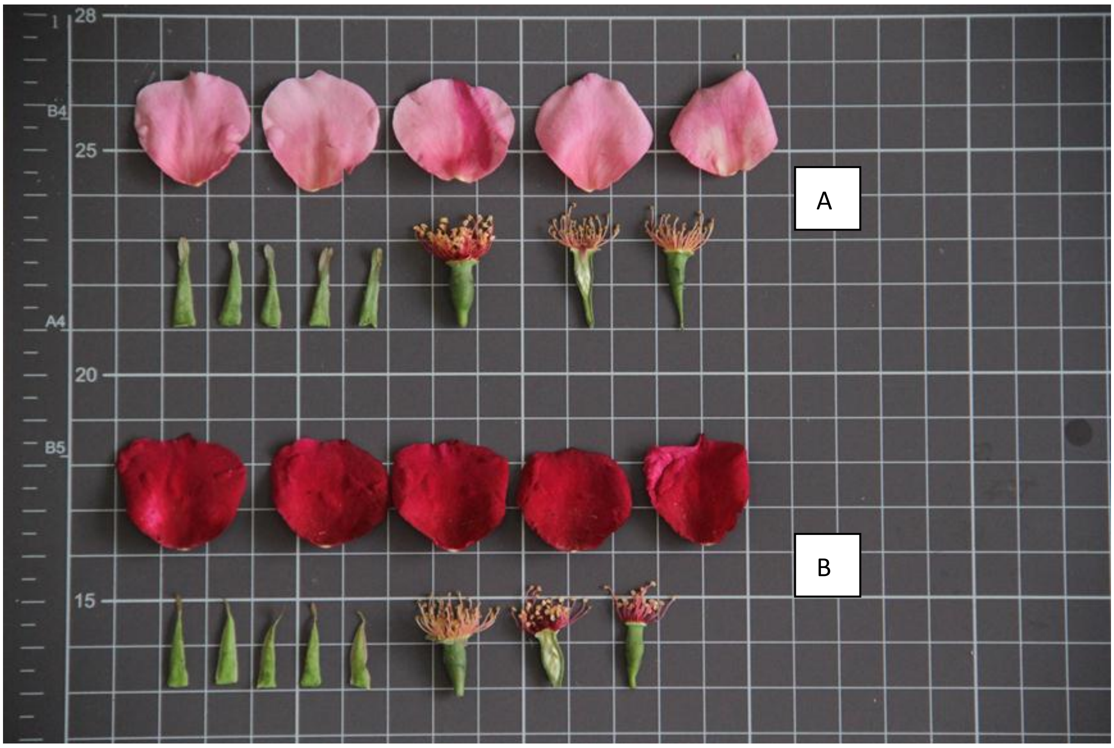

Figure S5. Flower organ dissection photos of *R. chinensis* var. *spontanea* from Nanjiang (A) and *R. lucidissima* from Anlong (B). Plants were propagated by cutting with stems collected from the wild and now growing in the Germplasm Nursery in Kunming.

Supplementary Tables

Table S1. Sequence polymorphisms detected in chloroplast DNA fragments *trnG-trnS* and *petL-psbE* of the *R. chinensis* var. *spontanea* and *R. lucidissima* species complex identifying 22 haplotypes (H1–H22)

Haplo type	trnS-trnG (1359bp)																		petL-petB(bp)																
	89	112	358	374	380	505	516	544	715	871	877	878	889	899	1087	1133	1137	1221	GeneBank No.	27	76	171	178	254	391	400	415	517	538	787	826	922	943	951	1133	GeneBank No.
H1	C	A	T	A	T	C	A	G	G	#1	–	–	–	#5	G	–	C	G	OL630606	G	–	C	T	#8	C	T	#9	C	–	T	T	C	G	A	T	OL630628
H2	C	A	T	A	T	C	A	A	G	#1	–	–	–	#5	G	–	C	G	OL630607	G	–	C	T	#8	C	T	#9	C	–	T	T	C	G	A	T	OL630629
H3	C	A	T	A	T	C	A	G	G	#1	G	#3	#4	#5	G	–	C	G	OL630608	A	#7	C	T	#8	C	T	#9	C	#10	T	T	C	T	A	T	OL630630
H4	C	A	T	A	T	C	A	G	G	#1	G	#3	#4	#5	G	–	C	G	OL630609	A	#7	C	T	#8	C	T	#9	C	#10	T	C	C	T	A	T	OL630631
H5	C	A	T	A	T	C	A	G	G	#1	–	–	–	#5	G	–	C	G	OL630610	A	#7	C	T	#8	C	T	#9	C	#10	T	T	G	T	A	T	OL630632
H6	C	A	T	A	G	C	A	G	G	#1	G	#3	#4	#5	G	–	C	G	OL630611	A	#7	C	T	#8	C	T	#9	C	#10	T	T	C	T	A	T	OL630633
H7	C	A	T	A	T	T	A	G	G	–	–	–	–	–	G	#6	A	G	OL630612	A	#7	C	G	–	A	T	#9	C	#10	T	T	C	G	A	G	OL630634
H8	C	A	C	A	T	T	A	G	G	#1	–	–	–	#5	T	#6	A	G	OL630613	A	#7	C	G	–	A	G	#9	C	#10	T	T	C	G	A	G	OL630635
H9	A	A	T	A	T	T	A	G	G	–	–	–	–	–	T	#6	A	A	OL630614	A	#7	C	T	–	A	T	#9	C	#10	T	T	C	G	C	T	OL630636
H10	C	A	T	A	T	C	A	G	G	#1	G	#3	#4	#5	G	–	C	G	OL630615	A	#7	C	T	#8	C	T	–	C	#10	T	T	C	T	A	T	OL630637
H11	C	A	T	A	T	C	A	G	G	#1	G	#3	#4	#5	G	–	C	G	OL630616	A	#7	C	T	–	C	T	–	C	#10	T	T	C	T	A	T	OL630638
H12	C	A	T	A	T	T	A	G	G	#1	T	#3	#4	#5	T	#6	A	G	OL630617	A	#7	C	G	–	A	T	#9	C	#10	T	T	C	G	A	G	OL630639
H13	C	A	T	A	T	T	A	G	G	#1	T	#3	#4	#5	G	#6	A	G	OL630618	A	#7	C	T	–	A	T	#9	C	#10	T	T	C	G	C	T	OL630640
H14	C	A	T	A	T	C	T	G	G	#1	–	–	–	–	T	–	C	G	OL630619	A	#7	A	T	–	A	T	#9	C	#10	T	T	C	G	A	T	OL630641
H15	C	A	T	C	T	C	A	G	G	#1	G	#3	#4	#5	G	–	C	G	OL630620	A	#7	C	T	#8	C	T	#9	C	#10	T	T	C	T	A	T	OL630642
H16	C	A	T	A	T	C	A	G	G	#1	G	#3	–	#5	G	–	C	G	OL630621	A	#7	C	T	#8	C	T	#9	C	#10	T	T	C	T	A	T	OL630643
H17	C	A	T	A	G	C	A	G	G	#1	–	–	–	#5	G	–	C	G	OL630622	G	–	C	T	#8	C	T	#9	C	#10	C	T	C	G	A	T	OL630644

H18	C	A	T	A	G	C	A	G	T	#1	G	#3	-	#5	G	-	C	G	OL630623	A	#7	C	T	#8	C	T	#9	C	#10	T	T	C	G	C	T	OL630645
H19	C	A	T	A	G	C	A	A	G	#1	-	-	-	#5	G	-	C	G	OL630624	G	-	C	T	#8	C	T	#9	C	#10	T	T	C	G	A	T	OL630646
H20	C	A	T	A	G	C	A	G	G	#1	-	-	-	#5	G	-	C	G	OL630625	G	-	C	T	#8	C	T	#9	C	#10	T	T	C	G	A	T	OL630647
H21	C	A	T	A	T	T	T	G	G	#1	-	-	-	#5	T	#6	A	G	OL630626	A	#7	C	G	-	A	T	#9	C	#10	T	T	C	G	A	G	OL630648
H22	C	C	T	A	T	C	A	G	G	#1	G	#3	#4	#5	G	-	C	G	OL630627	A	#7	C	T	#8	C	T	#9	T	#10	T	T	C	G	A	T	OL630649

Note： Dashes (-) means indel， #1=AAAAAA， #2=T/G， #3=AAATAACTAAA， #4=TAACAAAAAA， #5=TAAC， #6=CTT， #7=CAATTGAAAGGATAAGTTC， #8=TATACGTATATAACTAATATATGTATATTGAATAAACTAA， #9=ATTGAAAAA， #10=TATCACAT

Table S2. ITS Sequence polymorphisms detected in *R. chinensis* var. *spontanea* and *R. lucidissima* species complex identifying 21 haplotypes (rH1–rH21)

Haplotype	20	29	31	38	40	41	43	46	80	100	102	108	109	181	222	367	432	462	493	523	540	547	548	551	586	587	GenBank accession numbers
rH1	A	C	G	C	G	T	A	C	T	T	G	C	C	C	C	C	G	C	A	A	G	C	G	T	C	A	OL454760
rH2	G	C	G	C	G	T	A	C	T	T	G	C	C	C	C	C	G	C	A	A	G	C	G	T	C	A	OL454761
rH3	G	C	G	T	G	C	G	C	C	C	G	T	C	C	C	C	A	C	G	A	G	C	G	T	C	A	OL454762
rH4	G	C	G	T	T	C	G	C	T	T	A	C	C	C	C	C	A	C	G	A	G	C	G	T	C	C	OL454763
rH5	G	C	G	C	G	T	A	C	T	T	G	C	C	C	C	C	A	C	A	A	G	C	G	T	C	A	OL454764
rH6	G	T	T	T	G	C	G	C	C	C	G	T	C	T	C	T	G	C	G	G	G	T	C	T	C	A	OL454765
rH7	G	T	T	T	G	C	G	C	C	C	G	T	C	T	C	T	G	C	G	G	G	T	C	T	C	C	OL454766
rH8	G	C	G	C	G	T	A	C	T	T	G	C	C	C	C	C	G	T	G	A	G	C	G	T	T	C	OL454767
rH9	G	C	G	C	G	T	A	C	T	T	G	C	C	C	T	C	G	T	G	A	G	C	G	T	T	C	OL454768
rH10	G	C	G	C	G	T	A	T	T	T	A	C	C	C	T	C	G	C	G	A	G	C	G	T	T	C	OL454769
rH11	G	C	G	C	G	T	A	C	T	T	G	C	C	C	C	C	G	C	A	A	A	C	G	T	C	A	OL454770
rH12	G	C	G	T	T	C	G	T	T	T	A	C	C	C	C	C	A	C	G	A	G	C	G	T	C	C	OL454771
rH13	G	C	G	T	G	C	G	C	C	C	G	T	C	C	C	C	G	C	G	A	G	C	G	T	C	A	OL454772
rH14	G	C	G	T	T	C	G	T	T	T	A	C	C	C	C	C	G	C	G	A	G	C	G	T	C	C	OL454773

rH15	G	C	G	T	T	C	G	T	T	T	A	C	C	C	C	C	A	C	G	A	G	C	G	T	C	A	OL454774
rH16	G	C	G	C	G	T	A	C	T	T	G	C	C	T	C	T	G	C	G	G	G	C	G	T	C	A	OL454775
rH17	G	C	G	C	G	T	A	C	T	T	G	C	T	C	C	C	G	C	G	A	G	T	G	C	C	C	OL454776
rH18	G	C	G	T	G	C	G	C	C	C	G	T	C	C	C	C	A	C	G	A	G	C	G	T	C	C	OL454777
rH19	G	C	G	T	G	C	G	C	C	T	G	T	C	C	C	C	A	C	G	A	G	C	G	T	C	A	OL454778
rH20	G	C	G	T	G	C	G	C	C	C	G	T	C	C	C	C	G	C	G	A	G	C	G	T	C	C	OL454779
rH21	G	C	G	T	G	C	G	C	C	C	G	C	C	C	C	C	A	C	G	A	G	C	G	T	C	A	OL454780

Table S3. Results of AMOVA for cpDNA and ITS sequences variation of *R. chinensis* var. *spontanea* -*R. lucidissima* complex

cpDNA					
Dataset	Source of variation	d. f.	Sum of squares	Variance components	Percentage of variation
Species complex	Among populaitons	24	731.748	2.31278 Va	92.29
	Within populations	305	58.928	0.19321 Vb	7.71
	Total	329	790.676	2.50599	
Fixation Index F_{ST} : 0.92290					
R. *chinensis* var. *spontanea*	Among populaitons	15	224.613	1.08770 Va	89.54
	Within populations	205	26.048	0.12706 Vb	10.46
	Total	220	250.661	1.21476	
Fixation Index F_{ST} : 0.89540					
R. lucidissima	Among populaitons	8	383.376	4.02145 Va	92.44
	Within populations	100	32.880	0.32880 Vb	7.56
	Total	108	416.257	4.35025	
Fixation Index F_{ST} : 0.92442					

			ITS		
	Among populations	24	660.551	1.58191 Va	58.90
Species complex	Within populations	396	437.178	1.10398 Vb	41.10
	Total	420	1097.729	2.68589	
Fixation Index F_{ST} : 0.58897					
R. *chinensis* var. *spontanea*	Among populations	15	286.490	1.10711 Va	55.72
	Within populations	251	220.798	0.87967 Vb	44.28
	Total	266	507.288	1.98679	
Fixation Index F_{ST} : 0.55724					
R. lucidissima	Among populations	8	146.224	1.00398 Va	40.22
	Within populations	145	216.380	1.49228 Vb	59.78
	Total	153	362.604	2.49626	
Fixation Index F_{ST} : 0.40219					

Table S4. Nested contingency analysis of geographical associations and phylogeographical inferences made from a nested haplotype analysis of *R. chinenses* var. *spontanea* and *R. lucidissima* complex based on cpDNA and ITS

	cpDNA	
Clade	Clade Key	Inference
Clade 1-1	1-19 NO	Allopatric fragmentation.
Clade 1-5	1-2-3-4 NO	Restricted gene flow with isolation by distance.
Clade 2-12	1-19 NO	Allopatric fragmentation.
Clade 3-7	1-19 NO	Allopatric fragmentation.
Total Cladogram	1-2 IO	I-T Status Undetermined: Inconclusive outcome.
	ITS	
Clade 1-3	1-2-3-5-6*-7-8 YES	Restricted gene flow/dispersal but with some long-distance dispersal over intermediate areas not occupied by the species; or past gene flow followed by extinction of intermediate populations.<br>Too few Clades: Insufficient genetic resolution to discriminate between range expansion/colonization and restricted dispersal/gene flow.<br>Question 7 and 8 try to distinguish between long vs. short distance movement
Clade 1-14	1-2 IO	I-T Status Undetermined: Inconclusive outcome
Clade 2-3	1-2 IO	I-T Status Undetermined: Inconclusive outcome
Total Cladogram	1-2 IO	I-T Status Undetermined: Inconclusive outcome.
